# Synthesis, Characterization and Cytotoxic Evaluation of New Pyrrolo[1,2-*b*]pyridazines Obtained via Mesoionic Oxazolo-Pyridazinones

**DOI:** 10.3390/ijms241411642

**Published:** 2023-07-19

**Authors:** Beatrice-Cristina Ivan, Stefania-Felicia Barbuceanu, Camelia Mia Hotnog, Octavian Tudorel Olaru, Adriana Iuliana Anghel, Robert Viorel Ancuceanu, Mirela Antonela Mihaila, Lorelei Irina Brasoveanu, Sergiu Shova, Constantin Draghici, George Mihai Nitulescu, Florea Dumitrascu

**Affiliations:** 1 Department of Organic Chemistry, Faculty of Pharmacy, “Carol Davila” University of Medicine and Pharmacy, 6 Traian Vuia Street, 020956 Bucharest, Romania; cristina.ivan@drd.umfcd.ro; 2 Center of Immunology, “Stefan S. Nicolau” Institute of Virology, Romanian Academy, 285 Mihai Bravu Ave., 030304 Bucharest, Romania; camelia.hotnog@virology.ro (C.M.H.); mirela.mihaila@virology.ro (M.A.M.); lorelei.brasoveanu@virology.ro (L.I.B.); 3 Department of Pharmaceutical Botany and Cell Biology, Faculty of Pharmacy, “Carol Davila” University of Medicine and Pharmacy, 6 Traian Vuia Street, 020956 Bucharest, Romania; octavian.olaru@umfcd.ro (O.T.O.); adriana.anghel@umfcd.ro (A.I.A.); robert.ancuceanu@umfcd.ro (R.V.A.); 4 Laboratory of Inorganic Polymers, “Petru Poni” Institute of Macromolecular Chemistry, Aleea Grigore Ghica Voda, 41A, 700487 Iasi, Romania; 5 Laboratory of Advanced Materials in Biofarmaceutics and Technics, Moldova State University, 2009 Chişinău, Moldova; 6 “Costin D. Nenitescu” Institute of Organic and Supramolecular Chemistry, Romanian Academy, 202B Splaiul Independenței, 060023 Bucharest, Romania; cst_drag@yahoo.com (C.D.); fdumitra@yahoo.com (F.D.); 7 Department of Pharmaceutical Chemistry, Faculty of Pharmacy, “Carol Davila” University of Medicine and Pharmacy, 6 Traian Vuia Street, 020956 Bucharest, Romania; george.nitulescu@umfcd.ro

**Keywords:** pyrrolo[1,2-*b*]pyridazines, mesoionic oxazolo-pyridazinones, dipolarophile alkynes, 3 + 2 cycloaddition, X-ray diffraction, cytotoxicity, antitumor activity

## Abstract

New pyrrolo[1,2-*b*]pyridazines were synthesized by 3 + 2 cycloaddition reaction between mesoionic oxazolo-pyridazinones and methyl/ethyl propiolate. The mesoionic compounds were generated in situ by action of acetic anhydride on 3(2*H*)pyridazinone acids obtained from corresponding esters by alkaline hydrolysis followed by acidification. The structures of the compounds were confirmed by elemental analyses and IR, ^1^H-NMR, ^13^C-NMR, and X-ray diffraction data. The regioselectivity of cycloaddition was evidenced by NMR spectroscopy and confirmed by X-ray analysis. The compounds were evaluated for their cytotoxicity on plant cells (*Triticum aestivum* L.) and crustacean animal cells (*Artemia franciscana* Kellogg and *Daphnia magna* Straus). The results indicated that the tested compounds exhibited low toxicity on the plant cell (IC_50_ values higher than 200 µM), while on Artemia nauplii no lethality was observed. Daphnia magna assay showed that pyrrolo[1,2-*b*]pyridazines **5a** and **5c** could exhibit toxic effects, whereas, for the other compounds, toxicity was low to moderate. Also, the cytotoxic effects of the compounds were tested on three human adenocarcinoma-derived adherent cell lines (colon LoVo, ovary SK-OV-3, breast MCF-7). The in vitro compound-mediated cytotoxicity assays, performed by the MTS technique, demonstrated dose- and time-dependent cytotoxic activity for several compounds, the highest anti-tumor activity being observed for **5a**, **2c,** and **5f**, especially against colon cancer cells.

## 1. Introduction

**Figure 1:**
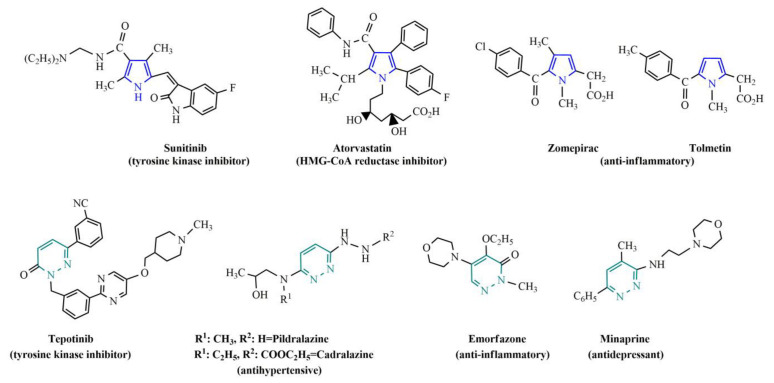
Structure of some drugs with pyrrole or pyridazine ring.

Five- and six-membered heterocyclic compounds present a special role in drug discovery design due to their remarkable biological properties [1,2,3]. From the five-membered heterocycles class, pyrroles are one of the most important compounds widespread in nature, being indispensable to life. The presence of the pyrrole nucleus in natural products (chlorophyll, heme B, bile pigments, etc.), in various drugs (e.g., sunitinib, atorvastatin, zomepirac, tolmetin) (Figure 1) and in numerous bioactive compounds with a wide spectrum of biological properties including antitumor action, is well known [4,5]. Six-membered heterocycles with pyridazine core are other natural or synthetic compounds, being a privileged scaffold in the development of new drugs, some of them being already marketed, such as tepotinib, pildralazine, cadralazine, emorfazone, minaprine (Figure 1) [6,7,8].

The interest in heterocyclic condensed systems with bridgehead nitrogen from pyrroloazines class has been growing for several decades, both for the versatility of their chemistry and especially for their pharmaceutical potential. From this class, pyrrolo[1,2-*b*]pyridazines, have particular interest owing to their biological activities and significant optical properties [9,10,11]. The chemistry and applications of pyrrolo[1,2-*b*]pyridazines have been reviewed extensively in 1977 [9] and then in 2008 [11]. Subsequently, new developments in the synthetic aspects [12,13,14,15] and applications of pyrrolo[1,2-*b*]pyridazines regarding their pharmacological potentials such as antitumor [16,17,18], antibacterial [19], anti-inflammatory [20,21,22], antidepressant [23], antimetabolic [24] actions and their optical properties were reported [25,26].

**Scheme 1:**
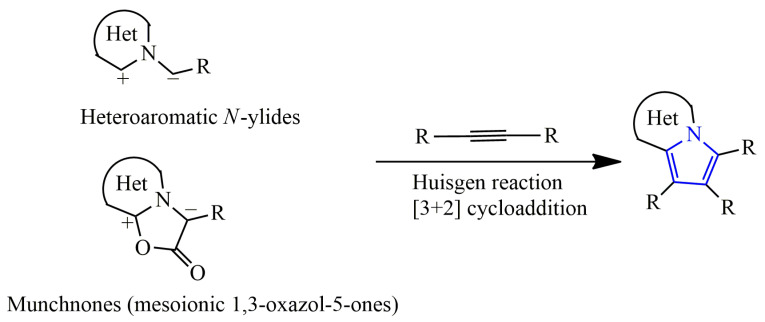
Two excellent routes for obtaining fused pyrroles including pyrrolo[1,2-*b*]pyridazines.

Regarding the synthesis of pyrroles and condensed pyrroles, the 1,3-dipolar cycloaddition reactions of heteroaromatic *N*-ylides [27,28,29] or mesoionic 1,3-oxazole-5-ones (münchnone) [30,31] with various acetylene or olefinic dipolarophiles proved to be a very useful method (Scheme 1). The cycloaddition reactions of the mesoionic münchnones is a chosen method in the synthesis of the compounds from these classes, this procedure has been applied by Dumitrascu et al. [32,33] for a new synthesis of pyrrolo[1,2-*b*]pyridazine derivatives, starting from 3(2*H*)pyridazinone acids. The pyridazinone skeleton is also present in many compounds with significant biological activities including anticancer, anti-inflammatory, analgesic, antimicrobial, antidiabetic, anticonvulsant, anxiolytic, antidepressant, antihypertensive [7,8,34,35,36,37], and optical properties [38,39].

The main methods leading to pyrrolo[1,2-*b*]pyridazines start from pyridazines or pyrroles. Two of the most productive methods starting from pyridazine, which involved the in situ generation of some *N*-ylides or mesoionic 1,3-oxazole-5-ones, are presented in Scheme 1.

Taking into account the literature data, herein we report the regioselective synthesis of new pyrrolo[1,2-*b*]pyridazines by 1,3-dipolar cycloaddition reaction between mesoionic bicyclic oxazolo-pyridazinones and non-symmetrical activated alkyne dipolarophiles. New pyrrolo[1,2-*b*]pyridazines and their acid precursors were investigated for their cytotoxic activity on plant cells (*Triticum aestivum* L.), crustacean cells (*Artemia franciscana* Kellogg and *Daphnia magna* Straus) and several human adherent cell lines derived from human solid tumors such as LoVo (colon adenocarcinoma), SK-OV-3 (ovary carcinoma), and MCF-7 (breast adenocarcinoma). The antiproliferative activity of the compounds under study was compared with the effects induced by several oncolytic drugs such as cisplatin (CisPt), doxorubicin (DOX), or 5-fluorouracil (5-FU), which were used as positive controls of the assays.

## 2. Results and Discussion

### 2.1. Chemistry

**Scheme 2:**
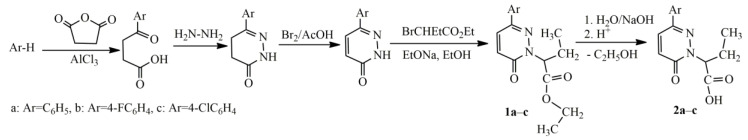
The synthesis of the 3(2*H*)pyridazinone-butanoic acids **2a**–**c** as precursors (starting materials) for pyrrolo[1,2-*b*]pyridazines.

1,3-Oxazol-5-ones or münchnones are a mesoionic compound named after the city where they were discovered by Huisgen [40]. The most significant property of münchnones is the 3 + 2 cycloaddition reaction with dipolarophiles giving various heterocyclic derivatives [30,31,40,41,42]. An illustrative example is the synthesis on the decagram scale of atorvastatin, one of the top-selling drugs, using as a key step the 1,3-dipolar cycloaddition between münchnones and acetylenic dipolarophiles [43]. Due to the low stability of some münchnones, the 3 + 2 cycloaddition reactions have been achieved by their in situ generation in the presence of the dipolarophile. It is known that in many cases the 1,3-dipolar cycloaddition reactions of münchnones with non-symmetrical acetylenic dipolarophiles are not completely regioselective. It was previously reported that the reaction between bicyclic oxazolo-pyridazinone münchnones and esters of acetylenedicarboxylic acid formed the corresponding pyrrolo[1,2-*b*]pyridazine derivatives [32,33]. Herein is investigated the regioselectivity and synthesis of the new pyrrolo[1,2-*b*]pyridazine derivatives by 3 + 2 cycloaddition between bicyclic oxazolo-pyridazinone münchnones and marginal acetylenic dipolarophiles such as methyl or ethyl propiolate. The starting material for in situ generation of bicyclic mesoionic münchnones **3a**–**c** was the 3(2*H*)pyridazinone-butanoic acid precursors **2a**–**c** that have been obtained by alkaline hydrolysis of corresponding esters **1a**–**c** followed by acidification, with good yields (87% for **2a** and 90% for **2b** and **2c**). The corresponding esters were synthesized from 6-aryl-3(2*H*)pyridazinone and ethyl 2-bromobutanoate by a procedure described in the literature for compounds with similar structure [44,45,46] (Scheme 2).

**Figure 2:**
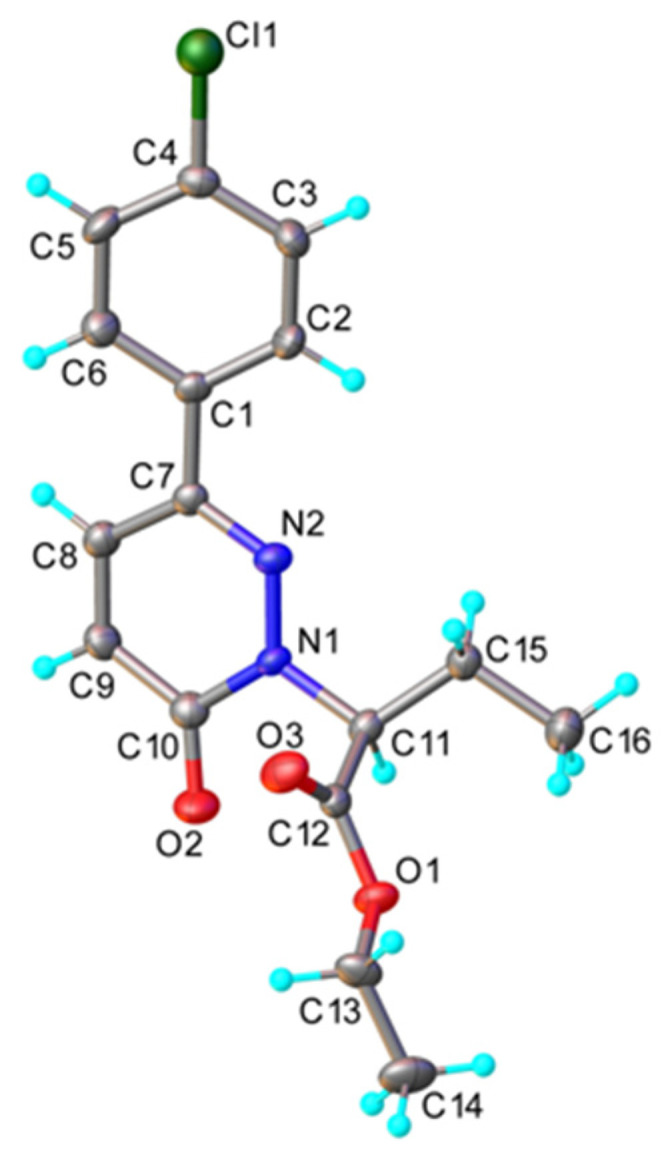
X-ray molecular structure of compound **1c** with atom labeling and thermal ellipsoids at 50% level.

**Figure 3:**
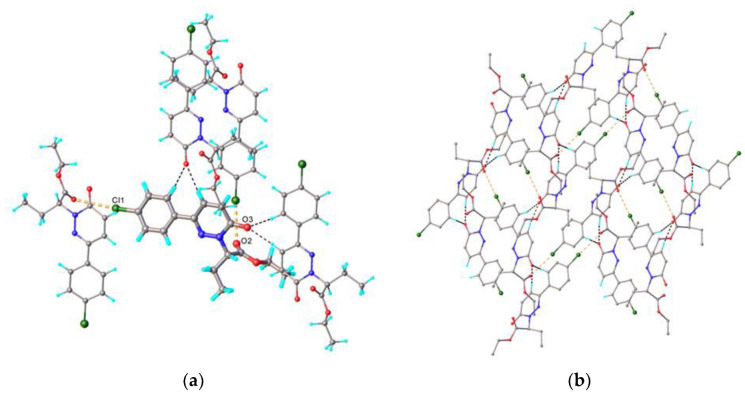
(**a**) View of the asymmetric unit in the crystal structure **1c** showing its interaction with adjacent molecules. Hydrogen and halogen bonds are shown as dotted lines with black and orange color, respectively. H-bonds parameters: C6-H···O3 [C6-H 0.95 Å, H···O3(1 − x, 0.5 + y, 1.5 − z) 2.51 Å, C6···O3 3.381(3) Å, ∠C6HO3 152.7°]; C8-H···O3 [C8-H 0.95 Å, H···O3(1 − x, 0.5 + y, 1.5 − z) 2.26 Å, C8···O3 3.205(3) Å, ∠C8HO3 175.8°]. Halogen bond parameters: [C4-Cl1···O2] C4-Cl1 1.739(3) Å, Cl1···O2(x, 1.5 − y, −0.5 + z) 3.133(2) Å, ∠C4Cl1O2 165.5(2)°; (**b**) A partial view of the crystal packing showing the formation of 3D supramolecular network in the crystal **1c**.

The structures of the esters **1a**–**c** were assigned by IR and NMR spectroscopy. The ^1^H-NMR spectra of these intermediates present as the main feature the absence of the NH signal of pyridazine moiety and the magnetic non-equivalence of the methylene protons from the CO_2_Et group which appear as a multiplet instead of a quartet due to the presence of the chiral carbon center in their molecule. The hydrogen atoms H-4 and H-5 from the pyridazinone core appear as two doublets with a coupling constant of 9.6 Hz, as expected. In the ^13^C-NMR spectra, the two types of carbonyl groups appear at *δ* between 159.7–160.1 ppm for the pyridazine moiety and 169.5–169.7 ppm for the ester group. The carbon signals of methyl from CO_2_C_2_H_5_ and -CHC_2_H_5_ groups appeared at 14 ppm and 10 ppm, respectively. Also, the -CH< chiral carbon signal is highlighted at 62.5 ppm while the methylene carbons from ester and alkyl groups resonated at 61.5 ppm and 23 ppm, respectively. The other carbon signals appeared at the corresponding chemical shifts. The most relevant absorption bands in the IR spectra are those corresponding to the stretching vibrations of the carbonyl group from lactam (ν_C=O_ = 1655–1665 cm^−1^) or ester (ν_C=O_ = 1734–1738 cm^−1^). The structure of the ester **1c** was confirmed by single crystal X-ray analysis, which has shown the compound to have a crystal structure comprising one molecular unit (Figure 2) and no co-crystallized interstitial molecules in the asymmetric part. The aromatic fragment is slightly non-planar with the dihedral angles between two rings of 18.13(8)°. Further analysis of the crystal packing has revealed the presence of short C-H···O and C-O···Cl contacts, which can be interpreted as intermolecular hydrogen and halogen bonding, respectively. As supramolecular aspects (Figure 3a) these contacts provide the direct interaction of each asymmetric unit with four adjacent molecules in the crystal. As a result, the crystal packing is characterized as a quite dense and complex three-dimensional network, as shown in Figure 3b.

**Figure 4:**
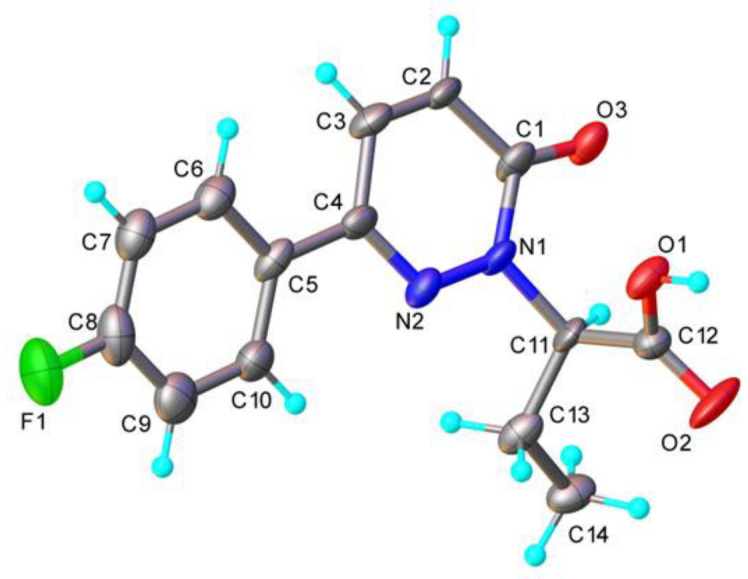
X-ray molecular structure of compound **2b** with atom labeling and thermal ellipsoids at 50% level.

**Figure 5:**
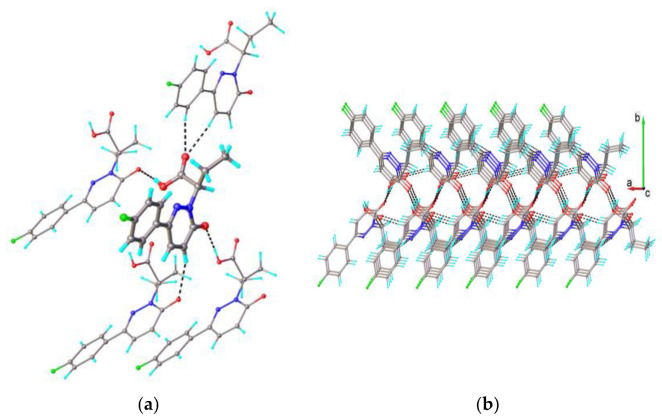
(**a**) View of the asymmetric unit in the crystal structure **2b** showing its interaction with adjacent molecules. Hydrogen bonds are shown as dotted lines with black color. H-bonds parameters: O1-H···O3 [O1-H 0.84 Å, H···O3(0.5 − x, 1.5 + y, z − 0.5) 1.73 Å, O1···O3 2.570(7) Å, ∠O1HO3 173.5°]; C2-H···O3 [C2-H 0.95 Å, H···O3(0.5 + x, 1.5 − y, 1.5 + z) 2.39 Å, C2···O3 3.268(8) Å, ∠C2HO3 154.3°]; C3-H···O2 [C3-H 0.95 Å, H···O2(1 + x, y, 1 + z) 2.40 Å, C3···O2 3.342(9) Å, ∠C3HO2 170.1°]; C14-H···N2 [C14-H 0.98 Å, H···N2(x − 1, y, z) 2.67 Å, C14···N2 3.59(1) Å, ∠C14HN2 156.4°]; (**b**) View of two-dimensional supramolecular layer in the crystal structure of compound **2b**.

The structures of the acids **2a**–**c** were also confirmed by NMR and IR spectroscopy. The proton and carbon NMR spectra are similar to those of the ester precursors. The disappearance of the protons and carbons signals of ethyl ester group from the NMR spectra of **2a**–**c** is the best proof that the hydrolysis took place. The chemical shifts for the carbonyl groups from the pyridazinone ring are close to those of the esters and appear in the range of 160.9–162.5 ppm, although NMR spectra were recorded in different solvents. The signal of carbonyl groups from acids appeared at *δ* = 173.5–175.6 ppm, being more deshielded compared to the ester intermediates. In the IR spectra, the representative absorption bands are those of ν_C=O_ (1708 cm^−1^) and ν_OH_ (2455–2524 cm^−1^) from carboxyl group. The structure of the acid **2b** was also confirmed by X-ray diffraction (Figure 4). Two aromatic rings in molecule **2b** form a dihedral angle of 17.0(2)°, which resembles the value found for molecule **1c**. The structural units are interconnected through O-H···O, N-H···O and C-H···O hydrogen bonding, so that the asymmetric part is surrounded by four neighboring molecules, as shown in Figure 5a. In the crystal the neutral molecules are packed to form discrete two-dimensional supramolecular layers, which are running parallel to 010 plane (Figure 5b).

**Scheme 3:**
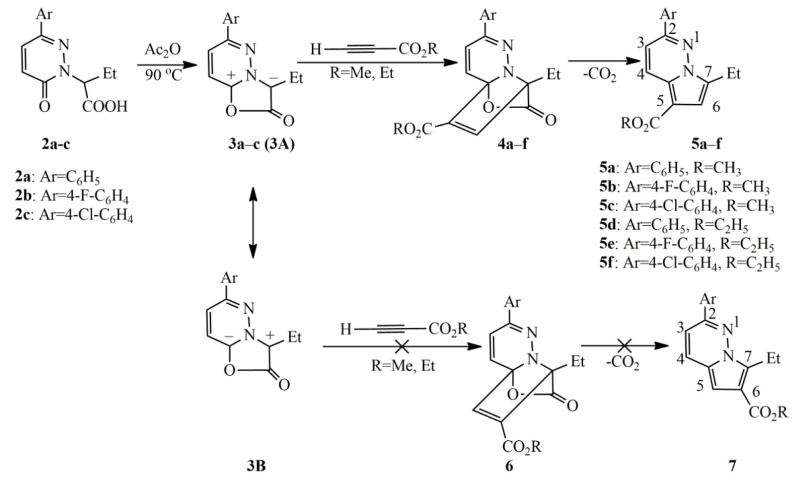
The regioselective synthesis of pyrrolo[1,2-*b*]pyridazines **5** from pyridazinone acids **2a**–**c**.

The new pyrrolo[1,2-*b*]pyridazine derivatives **5a**–**f** were obtained by 1,3-dipolar cycloaddition reactions between the bicyclic mesoionic 1,3-dipoles **3a**–**c** and methyl or ethyl propiolate as non-symmetrical acetylenic dipolarophiles, with yields between 41–52%. The mesoionic oxazolopyridazinones **3a**–**c** were in situ generated by the action of acetic anhydride on the acids **2a**–**c**. The generation of mesoionic 1,3-dipoles and cycloaddition reaction to form pyrrolo[1,2-*b*]pyridazines **5a**–**f** was performed in acetic anhydride at 90 °C for 3–4 h. The acetic anhydride was used both as reaction solvent and reagent which allows simultaneous dehydration and cyclization of pyridazinone acids **2a**–**c** to mesoionic compounds **3a**–**c** (Scheme 3).

The reaction mechanism of the obtaining of new pyrrolo[1,2-*b*]pyridazines **5a**–**f** implies in the first step the formation of mesoionic compounds **3** from acids **2**. The mesoionics **3** react as 1,3-dipoles **3A** with acetylenic dipolarophiles giving tricyclic intermediates **4** which, in the reaction conditions, eliminate carbon dioxide resulting in the corresponding pyrrolo[1,2-*b*]pyridazines having the ester group in the 5 position. The formation of the regioisomeric pyrrolo[1,2-*b*]pyridazines **7** from mesomeric form **3B** and intermediates **6** was not observed by NMR analysis of the crude reaction product. The regioselectivity of cycloaddition reaction and structures of the new compounds **5a**–**f** were assigned by NMR and IR spectroscopy and confirmed by X-ray diffraction for the representative compound **5a**.

The ^1^H-NMR data confirm the proposed regioselectivity of 3 + 2 cycloaddition, the ester groups of cycloadducts being in the 5 position of the pyrrolo[1,2-*b*]pyridazine ring. In diluted solutions, the pyrrolic proton H-6 appears as a triplet due to the coupling with methylenic protons of the ethyl group in the 7 position (*J*_H6-CH2_~0.9 Hz). The multiplicity of pyrrolic proton indicated that the only possible position for the hydrogen atom is in the 6 position of the pyrrolopyridazine ring. In the case when the cycloaddition had reversed regiochemistry, the pyrrolic proton would be in the 5 position and the coupling would not occur. Also, the absence in the spectra of these new compounds of the CH proton signals from the butanoic acid fragment in the intermediate acids (5.53–5.66 ppm) confirms the cycloaddition reaction. The ^13^C-NMR spectra present the expected signals, the main feature being the chemical shifts attributed to the carbonyl carbon of the ester groups which are in the range 164.5–165.1 ppm. The signal for C-6 from the pyrrole ring chemical has chemical shifts of 112.6–112.8 ppm. Compared to the precursor acids, the IR spectra of pyrrolopyridazines reveal a single absorption band, in the region 1688–1666 cm^−1^, due to the stretching vibration of the C=O ester group.

**Figure 6:**
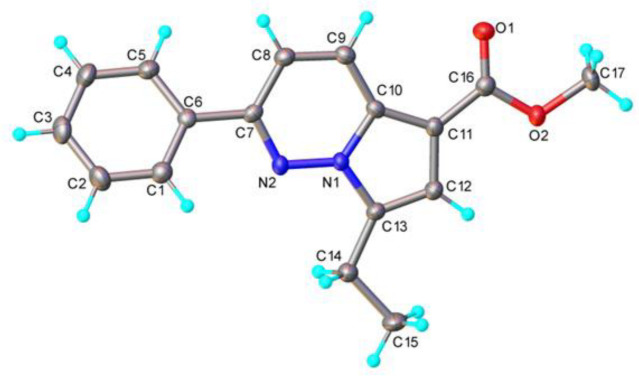
X-ray molecular structure of compound **5a** with atom labeling and thermal ellipsoids at 50% level.

**Figure 7:**
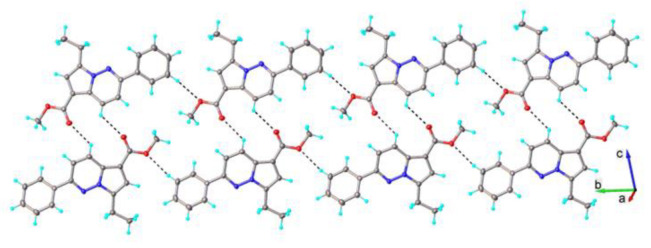
The role of hydrogen bonding in the formation of 1D supramolecular architecture in the crystal **5a**.

According to X-ray crystallography, compound **5a** crystallizes in the *P*-1 space group of the triclinic system with two chemically identic, but crystallographic independent molecules (denoted as **A** and **B**) in the asymmetric part of the unit cell. As an example, the structure of molecule A is depicted in Figure 6. Similar to compounds **1c** and **2b** the aromatic fragment in two independent molecules **A** and **B** is also non-planar with the dihedral angle of 26.82(6)° and 16.42(6)°, respectively. It is to note, that the main crystal packing motif is determined by a system of C-H···O hydrogen bonding and essentially arises from the parallel packing of a one-dimensional supramolecular array running along the *b* axis, as depicted in Figure 7.

Selected crystallographic data and structure refinement details for compounds **1c**, **2b,** and **5a** are provided in Table 1.

### 2.2. Toxicity Evaluation

#### 2.2.1. Plant Toxicity Assay

**Figure 8:**
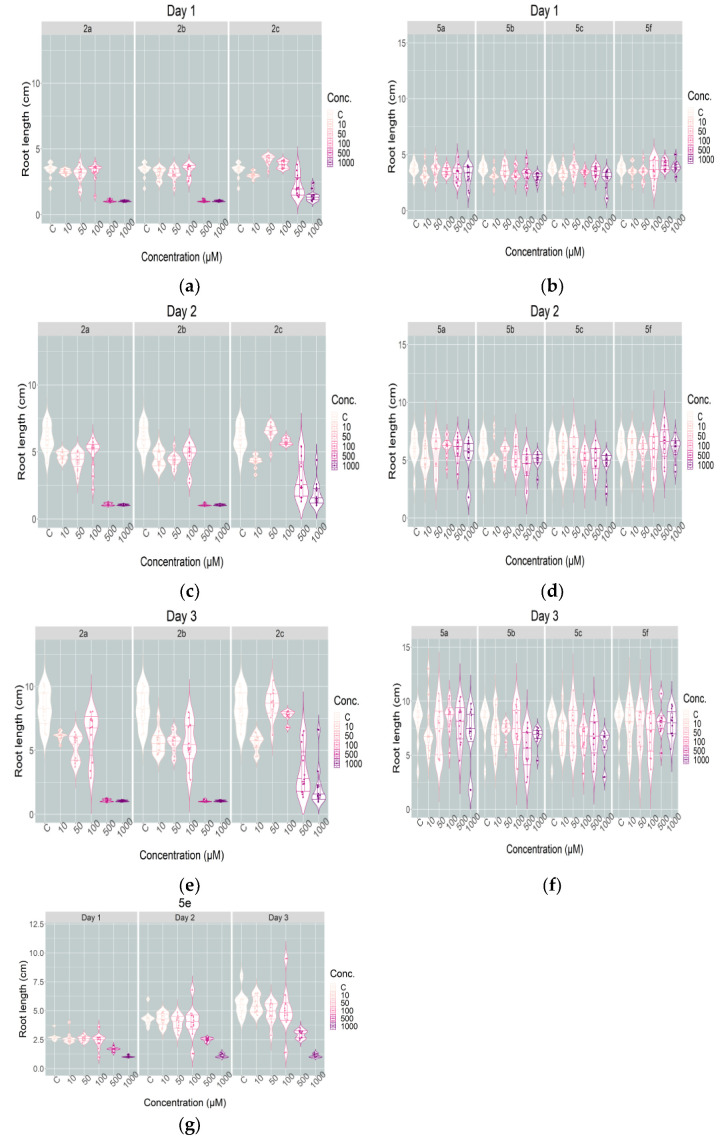
Violin plots showing the variations in Triticum’s main rootlet length under the influence of the tested compounds at different concentration levels and day of measurement: (**a**) day 1—acids **2a**–**b**; (**b**) day 1—pyrrolo[1,2-*b*]pyridazines **5a**–**c** and **5f**; (**c**) day 2—acids **2a**–**c**; (**d**) day 2—pyrrolo[1,2-*b*]pyridazines **5a**–**c** and **5f**; (**e**) day 3—acids **2a**–**c**; (**f**) day 3—pyrrolo[1,2-*b*]pyridazines **5a**–**c** and **5f**; (**g**) day 1, 2 and 3—pyrrolo[1,2-*b*]pyridazines **5e**.

**Figure 9:**
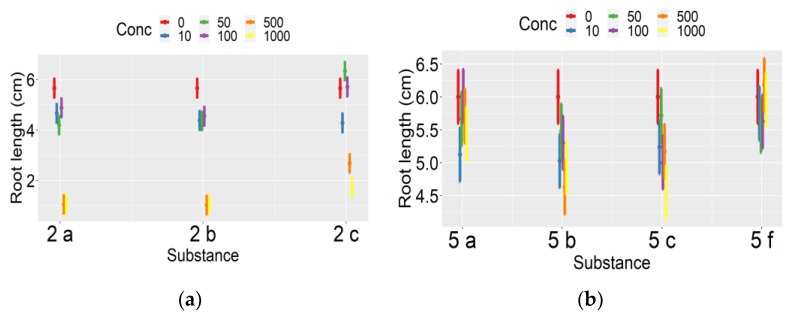
Interaction plot showing the interaction effects between the tested compounds and the concentration for the Triticum phytotoxicity test: (**a**)—acids **2a**–**c**; (**b**)—pyrrolo[1,2-*b*]pyridazines **5a**–**c** and **5f**.

The variation of rootlet lengths by compounds, concentration, and day of measurement are presented in Figure 8. There was good consistency between the parametric and robust mixed effects models with respect to the influence of the variables analyzed, but because of a number of outliers and considering the small differences, we here report the results for the robust model. As expected, root length increases with time (day of measurement) (*p* < 0.001) and there was generally a concentration-dependent inhibitory effect of the three first compounds (**2a**–**c**). There was no statistically significant difference between **2a** and **2b**, whereas for **2c** there were significant interactions between this derivative and concentration (particularly for the 500 μM—*p* < 0.001, 100 μM—*p* = 0.023, and 50 μM—*p* < 0.001 concentrations). The sense of the interactions is shown in Figure 9a. In the case of pyrrolo[1,2-*b*]pyridazines **5a**–**c** and **5f**, the root length also increased with time (day of measurement) (*p* < 0.001) (Figure 8). Considering a model that included interactions between compounds and concentration (as indicated by AIC to guide the model selection) there was no statistically significant main effects for these (*p* = 1.00), and for most concentrations, but for **5b** and **5c** there were several significant compound–concentration interactions (at 500 μM for **5b**, and 100 and 1000 μM for **5c**, *p* = 0.060, 0.010, and 0.023; the sense of the interactions is shown in Figure 9b. A simpler model, that excluded compound–concentration interactions (as suggested by using BIC instead of AIC to guide model selection), indicated that **5b** and **5c** had significantly stronger phytotoxic effects than **5a** (*p* < 0.001), whereas **5f** did not differ significantly from **5a** (*p* = 0.121). The compound **5e** was analyzed alone; therefore, it was only compared with the control group. It showed a concentration-dependent inhibitory effect, statistically significant at the first two levels (1000 and 500 μM, respectively—*p* < 0.001), approaching the conventional significance threshold at 100 μM (*p* = 0.059) and not significant at the lower concentrations (*p* > 0.145) (Figure 8g).

The IC_50_ values for those four compounds for which they could be estimated (monotonic concentration-dependent root length) are shown in Table 2. Based on the IC_50_ value, **2b** was the most phytotoxic (although the difference is small as compared with **2a**), whereas **5e** was the least phytotoxic. However, **5a**–**c** and **5f** seemed even less phytotoxic (IC_50_ values could not be estimated for the latter, because of the absence of a monotonic relationship between concentration and root length—Figure 8).

**Figure 10:**
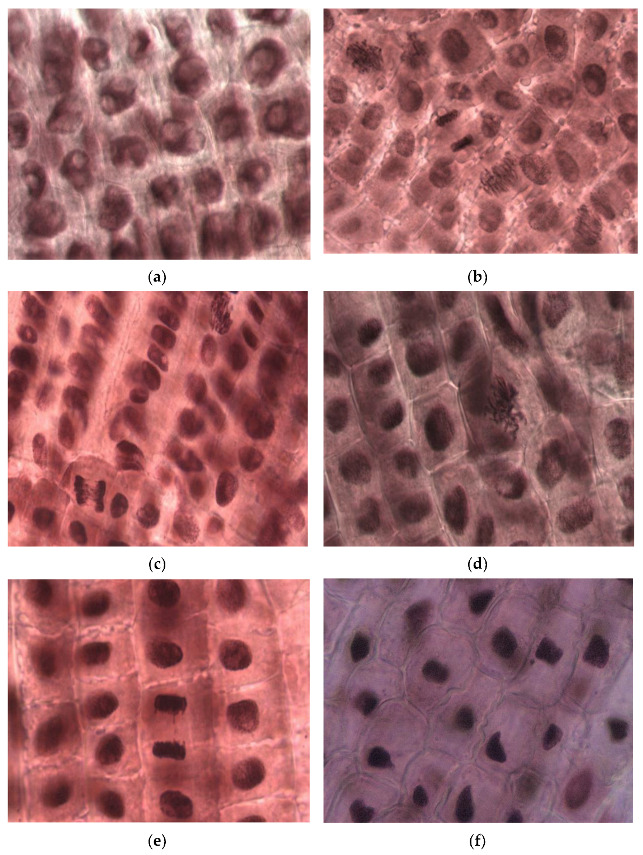
(**a**) Mitoinhibition and cells with 1-3 nucleoli observed in rootlets treated with **2b** (1000 µM); (**b**) telophase in tropokinesis, normal, or disorganized metaphase (**5b**, 1000 µM); (**c**) bridged telophase, disorganized metaphase (**5a**, 100 µM); (**d**) disorganized metaphase (**2c**, 1000 µM); (**e**) telophase with delayed chromosome, wavy cell walls (**5a**, 1000 µM); (**f**) undulated cell walls and modified shaped nuclei (**5e**, 1000 µM). All microphotographs were taken with a lens magnified by 40×.

The microphotograph analysis showed that at the highest concentration tested (1000 μM) compounds **2a**–**c** and **5e** caused mitoinhibition, while compounds **5a**–**c** caused only some mitotic film modifications. Among these, we mention: the oblique migration of chromosomes in metaphase and telophase (tropokinesis), the appearance of chromosomal bridges, or delayed chromosomes. These changes were also determined by compounds **2a**–**c** and **5e** at lower concentrations tested. Although compound **5a** had no mitoinhibitory effect at 1000 μM, it did affect the cell walls, which had a wavy appearance. The same effect was observed for compound **5e**, also appearing changes in the shape of the nuclei appeared. The migration of some chromosomes into the telophase was also delayed (e.g., **2b**-1000 µM, **5b**-1000 µM, **5a**-100 µM, **2c**-1000 µM, **5a**-1000 µM, **5e**-1000 µM; Figure 10).

#### 2.2.2. Animal Toxicity Assay

##### *Artemia franciscana* Toxicity Assay

No lethality was observed on nauplii of *Artemia franciscana* Kellogg for all compounds assessed in concentrations up to 1000 µM, indicating a lack of acute toxicity of these compounds. Artemia nauplii are generally more sensitive to toxicants than rodents [47], and the fact that the compounds were devoid of lethality on nauplii indicates a low level of toxicity. For comparison purposes, for two known biocides—tetrakis(hydroxymethyl) phosphonium chloride (THPC) and trichloroisocyanuric acid (TCIC)—LC_50_ values lower than 1 μM were determined in Artemia nauplii at 24 and 48 h [48]. The Artemia findings are in agreement with those of the phytotoxicity tests, where IC_50_ values, when estimation was possible, were in the range of hundreds of μM (indicating low phytotoxicity).

##### *Daphnia magna* Toxicity Assay

**Figure 11:**
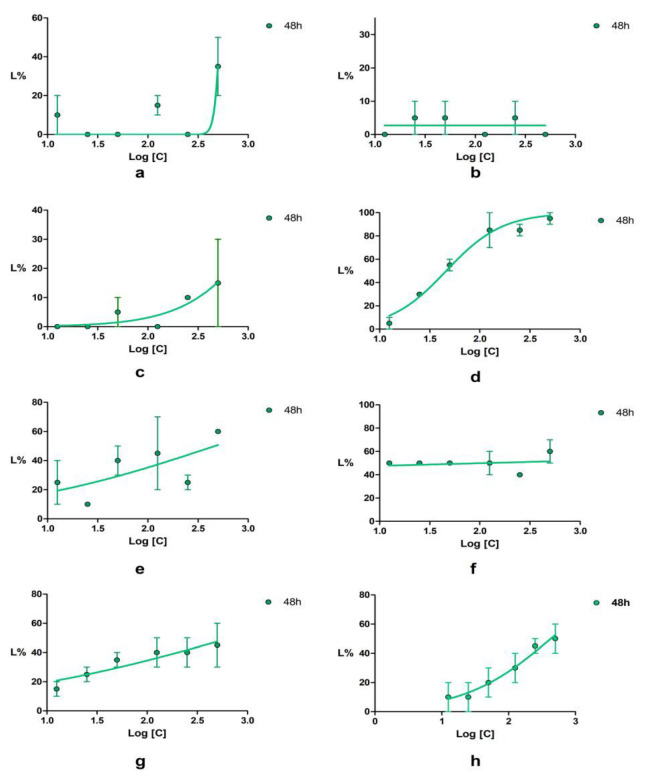
The lethality curves obtained after 48 h exposure of *Daphnia magna* to testing compounds. (**a**)—**2a**; (**b**)—**2b**; (**c**)—**2c**; (**d**)—**5a**; (**e**)—**5b**; (**f**)—**5c**; (**g**)—**5e**; (**h**)—**5f**; error bars represent the SD of two replicates.

At 24 h, except for compound **5a**, for which a maximum 40% lethality was obtained, the maximum average L% was 15%, data that are in accordance with the results obtained on *Artemia franciscana*. At 48 h, compounds **2b** and **2c** showed no toxicity, compounds **2a**, **5b**, **5e,** and **5f** exhibited moderate toxicity, whereas for **5a** LC_50_ was 46.12 µM and for **5c** was approximated to 106.8 µM. Though both compounds **5a** and **5c** exhibited toxicity, only for **5a** the concentration was correlated with the effect. The high differences between the L% induced by these two compounds could be attributed to the solubility, rather than the chemical difference (Table 3, Figure 11). The high sensibility of *Daphnia magna* versus *Artemia* sp. was also observed in our previous studies on compounds with pyrrole structure [49].

#### 2.2.3. Compound-Mediated Cytotoxicity Assays

The potential anti-proliferative effects of treatments with the new compounds under study were evaluated in vitro against solid tumor-derived cells of different histological origin vs. normal human endothelial cells. Therefore, several compound-mediated cytotoxicity assays were performed using three adherent tumor standardized cell lines derived from human colon adenocarcinoma LoVo [50,51,52,53], breast adenocarcinoma MCF-7 [49,54], and ovary adenocarcinoma SK-OV-3 [49,55], and compared to normal human umbilical vein endothelial cells (HUVEC) [56]. The cytotoxic activity of the newly synthesized compounds was compared to the one induced by cisplatin (Cis-Pt), 5-fluorouracil (5-FU), or doxorubicin (DOX), commonly used drugs for oncological treatments of cancers, and applied as positive controls throughout our experiments.

Thus, to discriminate between the compounds under study regarding their capacity to inhibit cell growth, tumor and normal cell cultures were treated with the new compounds or oncolytic drugs for 24 h or 48 h, and were further subjected to the MTS assay [52]; experimental data were calculated and percentages of cell viability were assessed for each compound under study.

**Figure 12:**
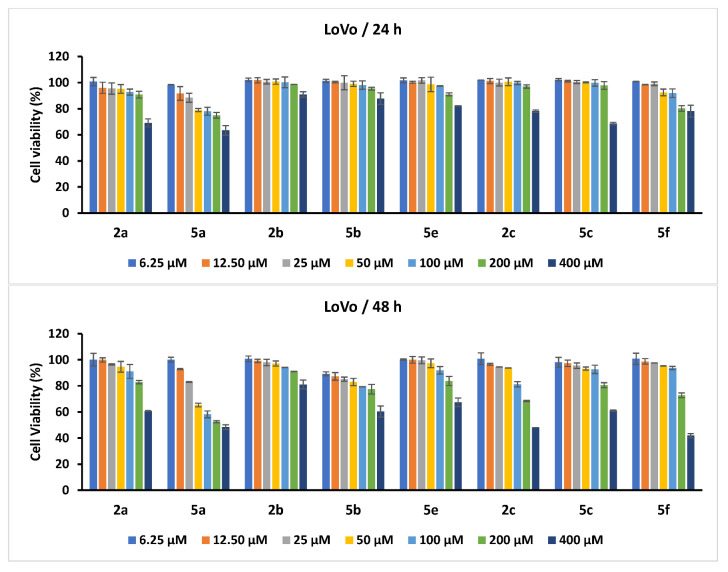
Anti-tumor effect of tested compounds against LoVo colon cancer cells. Viability of LoVo cells was measured after treatments with scalar concentrations of the compounds for 24 h or 48 h and compared to that of untreated control cells. Data shown are expressed as mean values ± standard deviations (SD) of three different experiments (n = 3).

**Figure 13:**
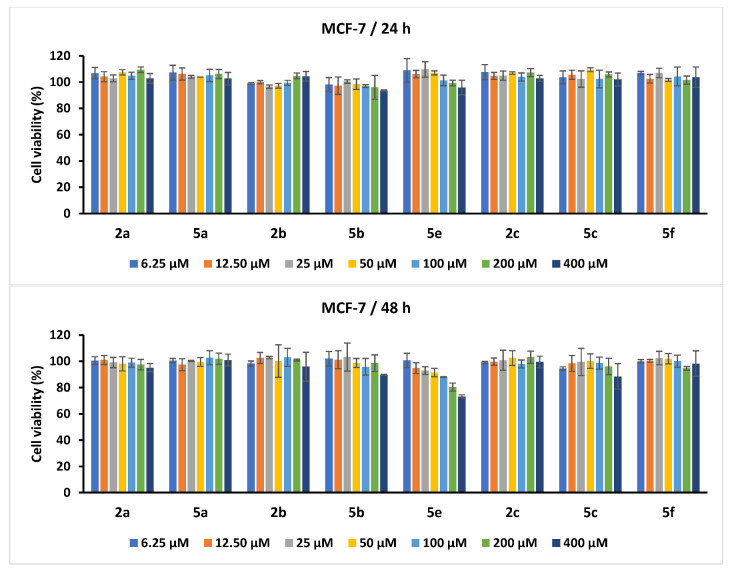
Anti-tumor effect of tested compounds against MCF-7 breast cancer cells. Viability of MCF-7 cells was measured after treatments with scalar concentrations of the compounds for 24 h or 48 h and compared to that of untreated control cells. Data shown are expressed as mean values ± standard deviations (SD) of three different experiments (n = 3).

**Figure 14:**
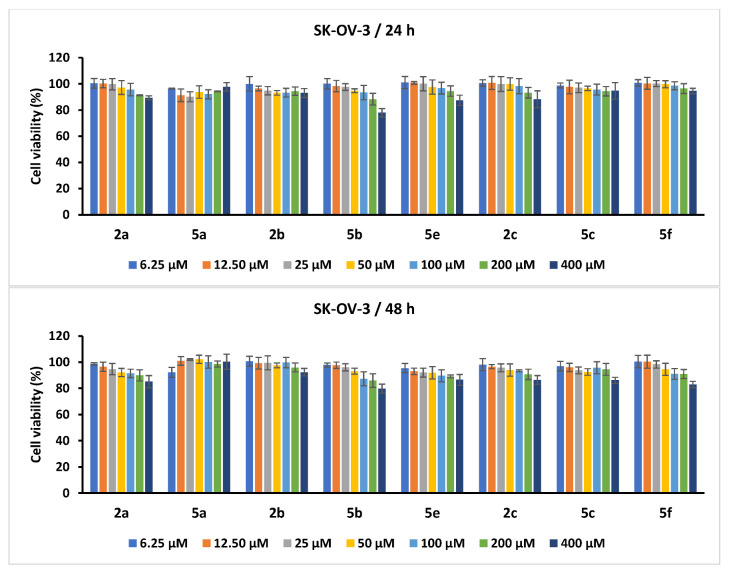
Anti-tumor effect of tested compounds against SK-OV-3 ovary cancer cells. Viability of SK-OV-3 cells was measured after treatments with scalar concentrations of the compounds for 24 h or 48 h and compared to that of untreated control cells. Data shown are expressed as mean values ± standard deviations (SD) of three different experiments (n = 3).

**Figure 15:**
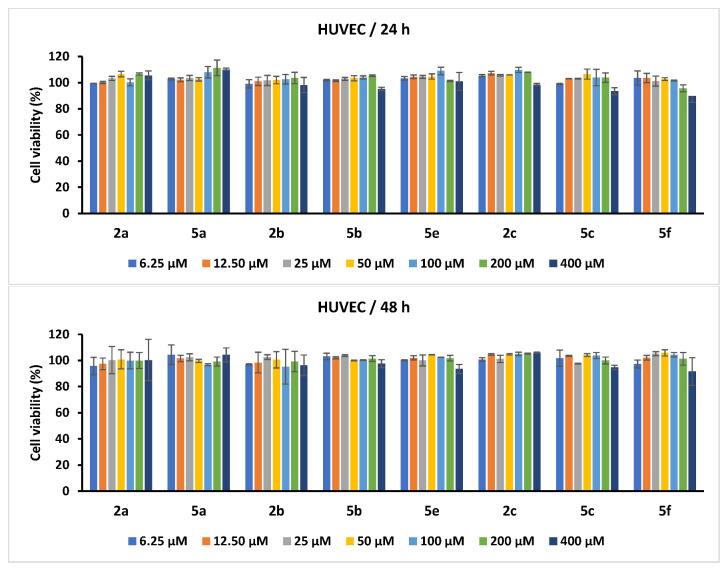
Anti-tumor effect of tested compounds against HUVEC endothelial cells. Cell viability was measured after HUVEC cell treatments with scalar concentrations of the compounds for 24 h or 48 h and compared to that of untreated control cancer cells. Data shown are expressed as mean values ± standard deviations (SD) of three different experiments (n = 3).

**Figure 16:**
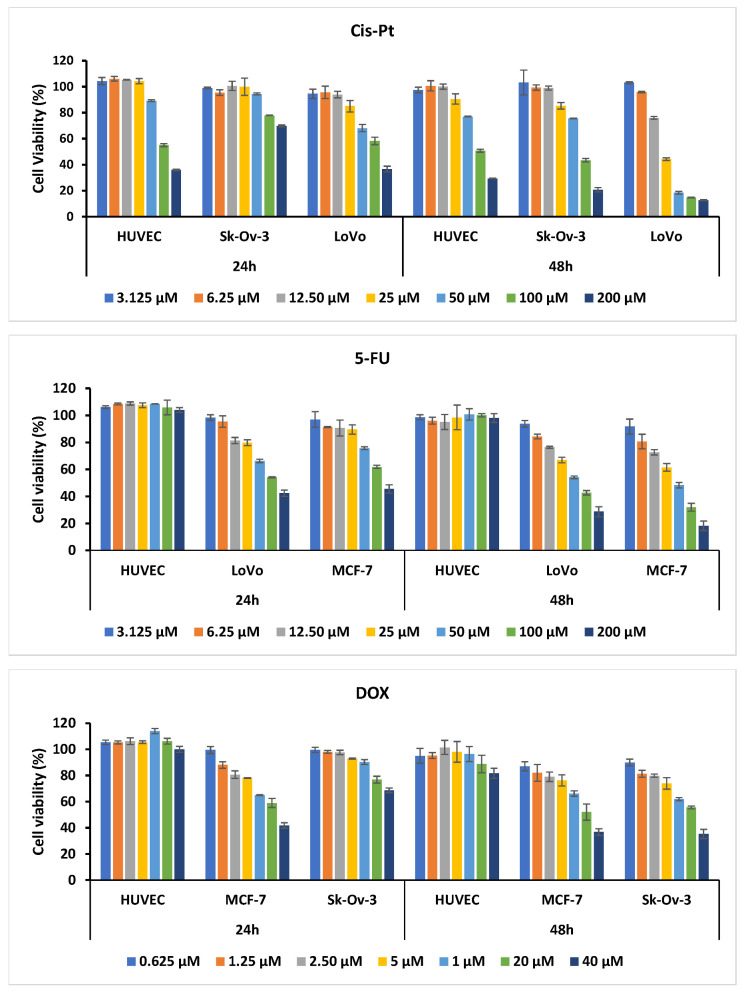
Anti-tumor effect of oncolytic control drugs against cancer vs. normal cells. The cell viability was measured after cell treatments with scalar concentrations of drugs for 24 h or 48 h and compared to that of untreated control cells. Data shown are expressed as mean values ± standard deviations (SD) of three different experiments (n = 3).

Therefore, the precursor acids **2a**–**c** and corresponding pyrrolo[1,2-*b*]pyridazines **5a**–**c**,**f** were tested for their potential cytotoxic activity. During the assays, increasing concentrations of the compounds, ranging from 6.25 to 400 mM, were added for 24 h or 48 h to cancer LoVo, SK-OV-3, MCF-7, and the reference HUVEC cells, previously cultured for 24 h in 96-well flat bottom plates. As positive controls of the tests, increasing concentrations of oncolytic drugs were also used: either 3.125 to 200 μM 5-FU and CisPt, or 0.625 to 40 μM DOX. Then, MTS reagent was added, and cells were incubated for 4 h at 37 °C in a 5% CO_2_ humidified atmosphere. The absorbance values were spectrophotometrically read to a Dynex ELISA reader at λ = 492 nM. The cytotoxic effects of the compounds under study varied depending on concentration, treatment time, and cell type, as shown in Figure 12, Figure 13, Figure 14, Figure 15 and Figure 16.

When the cell responses to compound treatments were analyzed and the percentages of cell viability were calculated for each compound and cell line, the strongest cytotoxic dose-dependent effects of the new compounds were observed against the LoVo colon cancer cell line. Thus, cell viability percentages decrease more after 48 h treatments when compared to 24 h ones, in a dose- and time-dependent manner.

The treatments with the highest concentration of 400 μM for 24 h induced the highest decreases in cell viability, under 80% for halogen-free acid **2a** (69.13%), and corresponding pyrrolo[1,2-*b*]pyridazine **5a** (63.46%), for **2c** containing a chlorine atom (78.17%) and its derivatives **5c** (68.56%) and **5f** (78.18%). Instead, fluorinated homologous **2b** and pyrrolo[1,2-*b*]pyridazine **5b** and **5e** were demonstrated to have a lower effect, and cell viability was reduced to 90.98%, 87.77%, and 82.24%, respectively. Even lower concentrations of **5a** inhibited the cell growth: both 50 and 100 μM, till 79%, and 78.1%, respectively (Figure 12).

When the treatment time of LoVo cells was prolonged to 48 h, the cytotoxic effects of the new compounds were increased. Concentrations of 50 μM and 100 μM of compound **5a** induced a decrease in cell viability to 65.35% and 58.18%, respectively, much lower than its precursor **2a**. When concentrations of **5a** were increased, the percentages of cell viability decreased more, to 52.48% for 200 μM, and 48.47% for 400 μM, compared to **2a**, which for the same concentrations achieved the cell viability of 82.88% and 60.76%, respectively. The same effect was observed for the derivatives **5b** and **5e** that demonstrated stronger anticancer effects than their precursor, **2b**: when 100 μM of **5b** were used, the cell viability percentages decreased to 79.44%. When the treatment of 400 μM was applied, a stronger inhibition of cell viability was observed, to 60.33% for **5b**, and 67.38% for **5e**, compared to 81.11% cell viability induced by the precursor **2b**.

The precursor **2c** and its pyrrolo[1,2-*b*]pyridazines **5c** and **5f** seem to have also good cytotoxic activity, the 400 μM treatments inhibiting the cell growth to 48.06%, 61.09%, and 41.82%, respectively (Figure 12).

Among the newly synthesized compounds under study, halogen-free acid **2a** and its pyrrolo[1,2-*b*]pyridazines **5a**, and acid **2c** containing a chlorine atom, and corresponding pyrrolo[1,2-*b*]pyridazines **5c** and **5f** demonstrated the strongest cytotoxic effects against LoVo tumor colon cells, dose-dependent, both for 24 h and 48 h, some of them inducing a decrease less than 50% of cell viability when the highest concentration was used (Figure 12). Thus, among the tested compounds containing a halogen atom grafted on the benzene ring, the presence of chlorine had a better effect on inhibiting the proliferation of LoVo cells compared to the fluorine atom.

When treatments with the compounds under study were applied in MCF-7 cell cultures, much lower inhibition of cell growth was observed, as compared to LoVo cells, both for 24 h and 48 h (Figure 13). A low anti-tumor effect measured through the cytotoxic activity was observed for **2a** and **5f**: when cells were treated for 48 h with 200 μM and 400 μM the cell viability percentages were between 97% and 94%, respectively, for both compounds. When treatment time was prolonged to 48 h, the cytotoxic effects of several compounds were amplified. The compound **5e** used in concentrations between 100–400 μM induced a decrease in cell viability of less than 90%. The strongest cytotoxic activity was obtained when cells were treated with 400 μM of the derivatives **5b**, **5e,** and **5c**, the treatments inducing a decrease in cells viability under to 89.7%, 73.13%, and 88.53%, respectively (Figure 13).

When the SK-OV-3 cells were subjected to treatments with the new compounds, the same low anti-proliferative and drug resistance profile was observed as in the MCF-7 cell line, both for 24 h and 48 h. However, when cells were treated for 24 h with 100 μM, the cell viability percentages decreased to 95% or less. The increase in the concentrations to 200 μM and 400 μM induced a higher inhibition of ovary cell growth. The strongest effect was observed following **2a**, **5b**, **5e,** and **2c** treatments with 400 μM that decreased the cell viability to 89.33%, 77.96%, 87.46%, and 88.30%, respectively (Figure 14).

When treatment time was prolonged till 48 h, cell viability percentages were slightly diminished following **2a**, **5e, 2c**, **5c**, and **5f** treatments, compared to 24 h incubation (Figure 14). The strongest cytotoxic effect seemed to be achieved by treatments with 400 μM of the above compounds, the percentages of cell viability decreasing to 85.14% and 83.02%, for **2a** and **5f**, respectively. Although compound **5b** had slightly less cytotoxicity at 48 h than at 24 h at 400 μM, it had the lowest percentages of cell viability at the highest concentrations (100 μM, 200 μM, 400 μM) compared to the other tested derivatives (Figure 14).

The normal HUVEC, used as reference cells of the assays, were treated for 24 h and 48 h with all the compounds under study, in the same experimental conditions as those performed on the cancer cell lines derived from various human solid adenocarcinomas. Treatments of HUVEC cells for 24 h with scalar concentrations of new compounds had no influence on cell growth or demonstrated low cytotoxicity, except **5f** when cells were treated by 400 μM and the cell viability percentages diminished to 90% (Figure 15). Even when the treatment time was prolonged till 48 h, the inhibition of cell growth did not increase, 400 μM of **5f** inducing a decrease in the cell viability to 91.57% (Figure 15). Therefore, the endothelial cells seemed not to be much affected by compound treatments, even used at high concentrations or prolonged time (Figure 15).

In addition, several specific oncolytic drugs, currently used in clinical treatments of solid tumors, were used throughout all the assays as positive controls. The percentages of cell viability decreased with the increase in the drug concentration, both for 24 h and 48 h treatments with 5-FU, CisPt, or DOX.

After 24 h treatments with the 200 μM of 5-FU and CisPt, the LoVo cell viability percentages decreased to 42.34% and 36.57%, while the prolonged time of treatment to 48 h increased the cytotoxic effect to 28.67% and 12.76% for 5-FU and CisPt, respectively, higher than the effects of **5a**, **2c**, or **5f** compounds that demonstrated the best antitumor activity against colon cells (Figure 12 and Figure 16).

Treatments of MCF-7 cells for 24 h with 200 μM of 5-FU or 40 μM of DOX demonstrated higher effects, 45.47% and 41.77%, respectively, of cell viability being achieved. After 48 h of drug treatments, the cell viability was decreased by 200 μM of 5-FU and 40 μM of DOX, to 18.21%, and 36.73%, respectively (Figure 16).

When ovary cancer cells SK-OV-3, known for their drug resistance to chemotherapeutic treatments, were subjected to 40 μM of DOX, and 200 μM of CisPt treatments for 24 h, a decrease in the cell viability was observed to 68.59% and 69.77%, respectively. The increase in the incubation time to 48 h with the same concentrations of DOX and CisPt diminished the cell viability to 35.23%, and 20.74%, respectively (Figure 16).

Both 24 h treatments of normal human umbilical vein endothelial cells HUVEC with 5-FU and DOX had no remarkable cytotoxic effects, while 100 μM and 200 μM treatments with CisPt induced a decrease in cell viability to 55% and 36%, respectively. When the incubation time increased to 48 h, the treatment with 40 μM of DOX diminished the percentages of cell viability to 81,68%, while 200 μM of 5-FU had no significant effect. In exchange, CisPt induced a decrease in the cell viability between 76.95% to 29.41% when used at concentrations ranging from 50 μM to 200 μM (Figure 16).

In terms of IC_50_ values, **5a**, **2c,** and **5f** displayed the best cytotoxic activities on LoVo cells, when treatment time was prolonged to 48 h (Table 4), and therefore these compounds might further be used in future functional studies on colon cancer cell lines.

### 2.3. Prediction of the Molecular Mechanism of Action

In order to better evaluate the antiproliferative effect of new 2-phenylpyrrolo[1,2-*b*]pyridazine and their 3(2*H*)pyridazinone acids derivatives, a PASS analysis was performed to indicate the post-probable biological targets for these. The analysis returned a number of 1789 possible targets for which the Pa values were higher than the corresponding Pi values. The Pa values were higher than 0.7 for only 19 targets. Of these targets, only the proteasome ATPase is correlated with anticancer effects. The use of proteasome inhibitors has proven to be clinically successful in treating various types of cancer, especially blood cancers. The proteasomal ATPase provides the energy for the substrate translocation and facilitates protein degradation [57]. This potential target was observed for the 3(2*H*)pyridazinone derivatives, and not for the structurally similar pyrrolo[1,2-*b*]pyridazine derivatives. The analysis of the predicted pharmacological effects revealed other ATPases as potential targets, like chloride-transporting ATPase, polyamine-transporting ATPase, phospholipid-translocating ATPase, proton-exporting ATPase, or myosin ATPase. These results indicate that the new compounds could function as a structural analog of ATP. This observation led to the search for other ATP-dependent targets and thus revealed significant Pa values for a few kinases, like sphinganine kinase, NADH kinase, and N-acylmannosamine kinase. Interestingly, the calculated Pa values were low in the case of protein kinases.

**Figure 17:**
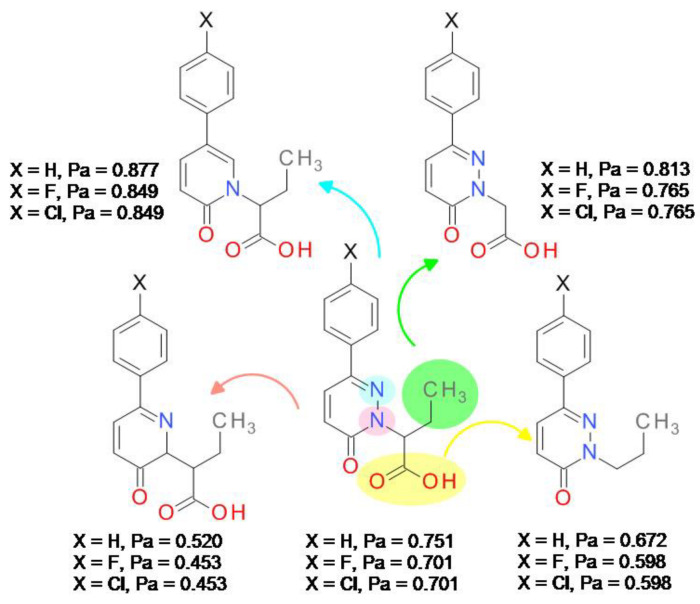
Analysis of the structural modifications on the potential to inhibit the proteasomal ATPase as predicted by PASS method.

We performed a series of theoretical structure modifications on the pyridazinone scaffold in order to observe the impact of various structural features on the compound’s potential to inhibit the proteasomal ATPase. The structures and the results are presented as Pa values in the following figure (Figure 17).

The structural modifications indicate that the presence of the halogen atom reduces the Pa values in all cases. The presence of the carboxyl group, as well as the presence of the 1-position nitrogen atom, seem to be important for the proteasomal ATPase interaction.

## 3. Materials and Methods

### 3.1. Chemistry

All reagents were of analytical grade and were purchased from commercial supplies (Sigma-Aldrich, Merck (Darmstadt, Germany), and Alfa Aesar (Haverhill, MA, USA)). The melting points, m.p., were determined on a Boëtius hot plate microscope (Carl Zeiss, Jena, Germany) and are uncorrected. The IR spectra were registered on a Vertex 70 spectrometer (Bruker Optik GmbH, Ettlingen, Germany) in ATR modes. The NMR spectra were recorded on a Varian Gemini 300BB spectrometer (Varian, Palo Alto, CA, USA) operating at 300 MHz for ^1^H and 75 MHz for ^13^C in CDCl_3_ or CDCl_3_ and TFA mixture as solvents, using TMS as the internal standard. The chemical shifts (*δ*) are reported in parts per million (ppm) and all coupling constants values *J* are given in hertz (Hz). The multiplicities are abbreviated as s—singlet, d—doublet, dd—doublet of doublets, t—triplet, q—quartet, qd—quartet of doublets, m—multiplet, b—broad. Single-crystal X-ray diffraction data were collected on an Oxford-Diffraction XCALIBUR Eos CCD diffractometer with graphite-monochromated Mo-Kα radiation. The unit cell determination and data integration were carried out using the CrysAlisPro package from Oxford Diffraction [58]. Multi-scan correction for absorption was applied. The structures were solved with program SHELXT using the intrinsic phasing method and refined by the full-matrix least-squares method on F2 with SHELXL [59,60]. Olex2 was used as an interface to the SHELX programs [61]. Non-hydrogen atoms were refined anisotropically. Hydrogen atoms were located in idealized positions and refined using a riding model (Supplementary Materials). The elemental analysis was achieved on a Costech Instruments EAS 32 (Costech Analytical Technologies, Valencia, CA, USA).

#### 3.1.1. General Procedure for the Synthesis of Esters **1a**–**c**

The esters **1a**–**c** were obtained from corresponding 3(2*H*)pyridazinone derivatives by an N-alkylation procedure described in the literature by McMillan and King [44,45].

Ethyl 2-(6-oxo-3-phenylpyridazin-1-yl)butanoate (**1a**). The compound was purified by crystallization from ethanol as colorless crystals with mp 56–58 °C; Yield 67%. Anal. Calcd. for C_16_H_18_N_2_O_3_ (286.33 g/mol): C, 67.12; H, 6.34; N, 9.78. Found C, 67.43; H, 6.72; N, 10.11. IR (ATR solid, cm^−1^): 1656 (ν_C=O_), 1735 (ν_C=O_), 3057 (ν_CH_). ^1^H-NMR (300 MHz, CDCl_3_) δ ppm: 0.98 (t, 3H, *J* = 7.4 Hz, CH_3_), 1.24 (t, 3H, *J* = 7.1 Hz, CH_3_), 2.32 (quintet, 2H, *J* = 7.4, 14.8 Hz, CH_2_), 4.17–4.27 (m, 2H, CH_2_O), 5.53 (t, 1H, *J* = 7.4 Hz, CH), 7.06 (d, 1H, *J* = 9.6 Hz, H-4), 7.42–7.45 (m, 3H, H-3′, H-4′, H-5′), 7.71 (d, 1H, *J* = 9.6 Hz, H-5), 7.77–7.80 (m, 2H, H-2′, H-6′). ^13^C-NMR (75 MHz, CDCl_3_) δ ppm: 10.7, 14.1 (2CH_3_), 23.0 (CH_2_), 61.5 (CH_2_O), 62.6 (CH), 125.9 (C-3′, C-5′), 128.9 (C-2′, C-6′), 129.5 (C-4′), 129.8 (C-4), 130.0 (C-5), 134.7 (C-1′), 144.3 (C-3), 160.1 (C-6), 169.7 (COO).

Ethyl 2-[6-oxo-3-(4-fluorophenyl)pyridazin-1-yl]butanoate (**1b**). The compound was purified by crystallization from ethanol as colorless crystals with mp 59–61 °C; Yield 72%. Anal. Calcd. for C_16_H_17_FN_2_O_3_ (304.32 g/mol): C, 63.15; H, 5.63; N, 9.21. Found C, 63.42; H, 5.94; N, 9.39. IR (ATR solid, cm^−1^): 1655 (ν_C=O_), 1734 (ν_C=O_), 3056 (ν_CH_). ^1^H-NMR (300 MHz, CDCl_3_) δ ppm: 0.98, (t, 3H, *J* = 7.4, Hz, CH_3_), 1.23 (t, 3H, *J* = 7.1 Hz, CH_3_), 2.23–2.36 (m, 2H, CH_2_), 4.13–4.26 (m, 2H, CH_2_O), 5.49, 5.54 (2d, 1H, *J* = 6.8 Hz, CH), 7.05 (d, 1H, *J* = 9.7, H-4), 7.14 (t, 2H, *J* = 8.9 Hz, H-3′, H-5′), 7.67 (d, 1H, *J* = 9.7 Hz, H-5), 7.77 (dd, 2H, *J* = 8.9, 5.2 Hz, H-2′, H-6′). ^13^C-NMR (75 MHz, CDCl_3_) δ ppm: 10.7, 14.1 (2CH_3_), 23.0 (CH_2_), 61.5 (CH_2_O), 62.6 (CH), 115.9 (d, 2C, *J* = 22.0 Hz, C-3′, C-5′), 127.7 (d, 2C, *J* = 8.6 Hz, C-2′, C-6′), 129.9 (C-4), 129.7 (C-5), 130.8 (d, *J* = 3.3 Hz, C-1′), 143.4 (C-3), 159.8 (C-6), 165.2 (d, *J* = 248.7 Hz, C-4′); 169.6 (COO).

Ethyl 2-[6-oxo-3-(4-chlorophenyl)pyridazin-1-yl]butanoate (**1c**). The compound was purified by crystallization from 2-propanol as colorless crystals with mp 49–51 °C; Yield 74%. Anal. Calcd. for C_16_H_17_ClN_2_O_3_ (320.77 g/mol): C, 59.91; H, 5.34; N, 8.73. Found C, 60.22; H, 5.72; N, 8.97. IR (ATR solid, cm^−1^): 1665 (ν_C=O_), 1738 (ν_C=O_), 3061 (ν_CH_). ^1^H-NMR (300 MHz, CDCl_3_) δ ppm: 0.98 (t, 3H, *J* = 7.4 Hz, CH_3_), 1.24 (t, 3H, *J* = 7.1 Hz, CH_3_), 2.23–2.36 (m, 2H, CH_2_), 4.17–4.27 (m, 2H, CH_2_O), 5.51, 5.54 (2d, 1H, *J* = 6.6 Hz, CH), 7.05 (d, 1H, *J* = 9.6 Hz, H-4), 7.42 (d, 2H, *J* = 8.3 Hz, H-3′, H-5′), 7.69 (d, 1H, *J* = 7.1 Hz, H-5), 7.73 (d, 2H, *J* = 8.3 Hz, H-2′, H-6′). ^13^C-NMR (75 MHz, CDCl_3_) δ ppm: 10.6, 14.0 (2CH_3_), 22.9 (CH_2_), 61.4 (CH_2_O), 62.5 (CH), 127.0 (C-3′, C-5′), 129.0 (C-2′, C-6′), 129.6 (C-4), 129.8 (C-5), 133.0 (C-4′), 135.5 (C-1′), 143.1 (C-3), 159.7 (C-6), 169.5 (COO).

#### 3.1.2. General Procedure for the Synthesis of Acids **2a**–**c**

The acids **2a**–**c** were obtained from alkaline hydrolysis of the corresponding esters by a procedure described in the literature by McMillan and King [44,45]. A mixture of the corresponding pyridazinone ester **1a**–**c** (50 mmol) and 100 mL of 10% sodium hydroxide solution was refluxed for 2 h. The reaction mixture was subsequently acidified at pH = 2 with 10% hydrochloric acid solution and the colorless precipitate formed was filtered off, washed with water, and then dried and purified by crystallization from a suitable solvent.

2-(6-Oxo-3-phenylpyridazin-1-yl)butanoic acid (**2a**). The compound was purified by crystallization from nitromethane as colorless crystals with mp 141–142 °C; Yield 87%. Anal. Calcd. for C_14_H_14_N_2_O_3_ (258.27 g/mol): C, 65.11; H, 5.46; N, 10.85. Found C, 65.39; H, 5.72; N, 11.14. IR (ATR solid, cm^−1^): 1632 (ν_C=O_), 1708 (ν_C=O_), 2932 (ν_OH_), 3061 (ν_CH_). ^1^H-NMR (300 MHz, CDCl_3_) δ ppm: 0.97 (t, 3H, *J* = 7.4 Hz, CH_3_), 2.28–2.42 (m, 2H, CH_2_), 5.53, 5.56 (2d, 1H, *J* = 6.4 Hz, CH), 7.16 (d, 1H, *J* = 9.6 Hz; H-4); 7.43–7.45 (m, 3H, H-3′, H-4′, H-5′), 7.71 (d, 1H, *J* = 9.6 Hz, H-5), 7.75–7.78 (m, 2H, H-2′, H-6′), 9.65 (bs, 1H, COOH). ^13^C-NMR (75 MHz, CDCl_3_) δ ppm: 10.8 (CH_3_), 22.9 (CH_2_), 63.2 (CH), 126.2 (C-3′, C-5′), 129.1 (C-2′, C-6′), 129.8 (C-4′), 129.9 (C-4), 130.6 (C-5), 134.6 (C-1′), 145.2 (C-3), 160.9 (C-6), 173.5 (COO).

2-[6-Oxo-3-(4-fluorophenyl)pyridazin-1-yl]butanoic acid (**2b**). The compound was purified by crystallization from nitromethane as colorless crystals with mp 174–175 °C; Yield 90%. Anal. Calcd. for C_14_H_13_FN_2_O_3_ (276.26 g/mol): C, 60.87; H, 4.74; N, 10.14. Found C, 61.18; H, 5.05; N, 10.41. IR (ATR solid, cm^−1^): 1633 (ν_C=O_), 1708 (ν_C=O_), 2980 (ν_OH_), 3073 (ν_CH_). ^1^H-NMR (300 MHz, CDCl_3_ + TFA) δ ppm: 1.03 (t, 3H, *J* = 7.4 Hz, CH_3_), 2.38–2.49 (m, 2H, CH_2_), 5.64, 5.66 (2d, 1H, *J* = 6.6 Hz, CH), 7.21 (t, 2H, *J* = 8.3 Hz, H-3′, H-5′), 7.47 (d, 1H, *J* = 9.6 Hz, H-4), 7.82 (dd, 2H, *J* = 9.0, 5.1 Hz, H-2′, H-6′), 7.98 (d, 1H, *J* = 9.6 Hz, H-5). ^13^C-NMR (75 MHz, CDCl_3_ + TFA) δ ppm: 10.4 (CH_3_), 22.7 (CH_2_), 64.2 (CH), 116.4 (d, 2C, *J* = 20.4 Hz, C-2′, C-6′), 128.9 (C-4), 132.3 (C-5), 128.4 (d, *J* = 8.6 Hz, C-2′, C-6′), 129.5 (d, *J* = 3.4 Hz, C-1′); 147.5 (C-3), 162.4 (C-6), 164.1 (d, *J* = 250.2 Hz, C-4′), 175.6 (COO).

2-[6-Oxo-3-(4-chlorophenyl)pyridazin-1-yl]butanoic acid (**2c**). The compound was purified by crystallization from ethanol as colorless crystals with mp 191–193 °C; Yield 90%. Anal. Calcd. for C_14_H_13_ClN_2_O_3_ (292.72 g/mol): C, 57.44; H, 4.48; N, 9.57. Found C, 57.70; H, 4.79; N, 9.80. IR (ATR solid, cm^−1^): 1631 (ν_C=O_), 1708 (ν_C=O_), 2931 (ν_OH_), 3079 (ν_CH_). ^1^H-NMR (300 MHz, CDCl_3_ + TFA) δ ppm: 1.03 (t, 3H, *J* = 7.4 Hz, CH_3_), 2.38–2.50 (m, 2H, CH_2_), 5.61, 5.63 (2d, 1H, *J* = 6.7 Hz, CH), 7.45 (d, 1H, *J* = 9.6 Hz, H-4), 7.48 (d, 2H, *J* = 8.7 Hz, H-3′, H-5′), 7.77 (d, 2H, *J* = 8.7 Hz, H-2′, H-6′), 7.97 (d, 1H, *J* = 9.6 Hz, H-5). ^13^C-NMR (75 MHz, CDCl_3_ + TFA) δ ppm: 10.4 (CH_3_), 22.7 (CH_2_), 64.4 (CH), 127.6 (C-3′, C-5′), 129.0 (C-4), 129.6 (C-2′, C-6′), 131.8 (C-1′), 132.3 (C-5), 137.2 (C-4′), 147.4 (C-3), 162.5 (C-6), 175.5 (COO).

#### 3.1.3. General Procedure for the Synthesis of New Methyl/Ethyl 7-Ethyl-2-arylpyrolo[1,2-b]pyridazine-5-carboxylate **5a**–**f**

The pyridazinone acids **2a**–**c** (15 mmol) were dissolved in 20 mL of hot acetic anhydride. Over the solution cooled to room temperature, 19 mmol of activated alkyne was added under stirring and the reaction mixture was heated at 90 °C for 3 h. The reaction mixture was cooled and then 30 mL of ethanol was added when a precipitate was obtained. The yellow fluorescent crystals were separated by filtration and were crystallized from alcohol (methanol, ethanol).

Methyl 7-ethyl-2-phenylpyrrolo[1,2-*b*]pyridazine-5-carboxylate (**5a**). The compound was purified by crystallization from methanol as fluorescent yellow crystals with mp 98–100 °C; Yield 42%. Anal. Calcd. for C_17_H_16_N_2_O_2_ (280.32 g/mol): C, 72.84; H, 5.75; N, 9.99. Found C, 73.13; H, 5.97; N, 10.28. IR (ATR solid, cm^−1^): 1685 (ν_C=O_), 3021 (ν_CH_). ^1^H-NMR (300 MHz, CDCl_3_) δ ppm: 1.41 (t, 3H, *J* = 7.1, 7.6 Hz, CH_3_), 3.03 (qd, 2H, *J* = 7.4, 0.9 Hz, CH_2_), 3.91 (s, 3H, CH_3_O), 7.11 (t, 1H, *J* = 0.9 Hz, H-6), 7.23 (d, 1H, *J* = 9.5 Hz, H-3), 7.45–7.48 (m, 3H, H-3′, H-4′, H-5′), 7.95–7.98 (m, 2H, H-2′, H-6′), 8.42 (d, 1H, *J* = 9.4 Hz, H-4). ^13^C-NMR (75 MHz, CDCl_3_) δ ppm: 11.7 (CH_3_), 18.8 (CH_2_), 51.0 (CH_3_O), 103.0 (C-5), 111.7 (C-3), 112.6 (C-6), 126.8 (C-2′, C-6′), 127.6 (C-4), 128.3, 132.9 (C-4a, C-7), 128.9 (C-3′, C-5′), 129.7 (C-4′), 136.2 (C-1′), 150.5 (C-2), 165.0 (COO).

Methyl 7-ethyl-2-(4-fluorophenyl)pyrrolo[1,2-*b*]pyridazine-5-carboxylate (**5b**). The compound was purified by crystallization from ethanol as fluorescent yellow crystals with mp 131–133 °C; Yield 41%. Anal. Calcd. for C_17_H_15_FN_2_O_2_ (298.31 g/mol): C, 68.45; H, 5.07; N, 9.39. Found C, 68.81; H, 5.33; N, 9.67. IR (ATR solid, cm^−1^): 1685 (ν_C=O_); 3058 (ν_CH_). ^1^H-NMR (300 MHz, CDCl_3_) δ ppm: 1.38 (t, 3H, *J* = 7.5 Hz, CH_3_), 3.01 (q, 2H, *J* = 7.5 Hz, CH_2_), 3.89 (s, 3H, CH_3_O), 7.09–7.17 (m, 4H, H-3, H-6, H-3′, H-5′), 7.93 (dd, 2H, *J* = 8.3, 5.4 Hz, H-2′, H-6′), 8.42 (d, 1H, *J* = 9.4 Hz, H-4). ^13^C-NMR (75 MHz, CDCl_3_) δ ppm: 11.9 (CH_3_), 19.0 (CH_2_), 51.3 (CH_3_O), 103.4 (C-5), 111.6 (C-3), 112.8 (C-6), 116.1 (2C, *J*_C-F_ = 22.0 Hz, C-2′, C-6′), 128.0 (C-4), 128.4, 133.1 (C-4a, C-7), 128.9 (d, 2C, *J*_C-F_ = 8.4 Hz, C-3′, C-5′), 132.5 (d, *J*_C-F_ = 2.8 Hz, C-1′), 149.8 (C-2), 162.5 (d, *J*_C-F_ = 249.4 Hz, C-4′), 165.1 (COO).

Methyl 2-(4-chlorophenyl)-7-ethylpyrrolo[1,2-*b*]pyridazine-5-carboxylate (**5c**). The compound was purified by crystallization from ethanol as fluorescent yellow crystals with mp 143–144 °C; Yield 52%. Anal. Calcd. for C_17_H_15_ClN_2_O_2_ (314.77 g/mol): C, 64.87; H, 4.80; N, 8.90. Found C, 64.59; H, 5.07; N, 9.13. IR (ATR solid, cm^−1^): 1681 (ν_C=O_); 3105 (ν_CH_). ^1^H-NMR (300 MHz, CDCl_3_) δ ppm: 1.40 (t, 3H, *J* = 7.5 Hz, CH_3_), 3.07 (q, 2H, *J* = 7.5 Hz, CH_2_), 3.90 (s, 3H, CH_3_O), 7.13 (s, 1H, H-6), 7.20 (d, 1H, *J* = 9.5 Hz, H-3), 7.45 (d, 2H, *J* = 8.6 Hz, H-3′, H-5′), 7.91 (d, 2H, *J* = 8.6 Hz, H-2′, H-6′), 8.46 (d, 1H, *J* = 9.4 Hz, H-4). ^13^C-NMR (75 MHz, CDCl_3_) δ ppm: 11.7 (CH_3_), 18.9 (CH_2_), 51.2 (CH_3_O), 103.4 (C-5), 111.3 (C-3), 112.8 (C-6), 128.0 (C-4), 128.1 (C-2′, C-6′), 128.3, 133.1 (C-4a, C-7), 129.2 (C-3′, C-5′), 134.7 (C-1′), 136.0 (C-4′), 149.5 (C-2), 165.0 (COO).

Ethyl 7-ethyl-2-phenylpyrrolo[1,2-*b*]pyridazine-5-carboxylate (**5d**). The compound was purified by crystallization from ethanol as fluorescent yellow crystals with mp 99–101 °C; Yield 41%. Anal. Calcd. for C_18_H_18_N_2_O_2_ (294.35 g/mol): C, 73.45; H, 6.16; N, 9.52. Found C, 73.78; H, 6.55; N, 9.77. IR (ATR solid, cm^−1^): 1666 (ν_C=O_); 3055 (ν_CH_). ^1^H-NMR (300 MHz, CDCl_3_) δ ppm: 1.41, 1.42 (2t, 6H, *J* = 7.1, 7.6 Hz, 2CH_3_), 3.07 (qd, 2H, *J* = 7.1, 0.9 Hz, CH_2_), 4.39 (q, 2H, *J* = 7.1 Hz, CH_2_O), 7.15 (t, 1H, *J* = 0.9 Hz, H-6), 7.26 (d, 1H, *J* = 9.5 Hz, H-3), 7.46–7.54 (m, 3H, H-1′, H-3′, H-5′), 7.98–8.02 (m, 2H, H-2′, H-6′), 8.48 (d, 1H, *J* = 9.4 Hz, H-4). ^13^C-NMR (75 MHz, CDCl_3_) δ ppm: 11.7, 14.6 (2CH_3_), 18.8 (CH_2_), 59.7 (CH_2_O), 103.4 (C-5), 111.7 (C-3), 112.6 (C-6), 126.8 (C-2′, C-6′), 127.8 (C-4), 128.3, 132.9 (C-4a, C-7), 128.9 (C-3′, C-5′), 129.7 (C-4′), 136.4 (C-1′), 150.6 (C-2), 164.6 (COO).

Ethyl 7-ethyl-2-(4-fluorophenyl)pyrrolo[1,2-*b*]pyridazine-5-carboxylate (**5e**). The compound was purified by crystallization from ethanol as fluorescent yellow crystals with mp 111–113 °C; Yield 47%. Anal. Calcd. for C_18_H_17_FN_2_O_2_ (312.34 g/mol): C, 69.22; H, 5.49; N, 8.97. Found C, 69.57; H, 5.81; N, 9.28. IR (ATR solid, cm^−1^): 1688 (ν_C=O_); 3073 (ν_CH_). ^1^H-NMR (300 MHz, CDCl_3_) δ ppm: 1.42 (t, 6H, *J* = 7.5 Hz, 2CH_3_), 3.07 (q, 2H, *J* = 7.5 Hz, CH_2_), 4.39 (q, 2H, *J* = 7.1 Hz, CH_2_O), 7.14–7.26 (m, 4H, H-3, H-6, H-3′, H-5′), 7.99 (dd, 2H, *J* = 8.8, 5.3 Hz, H-2′, H-6′), 8.49 (d, 1H, *J* = 9.4 Hz, H-4). ^13^C-NMR (75 MHz, CDCl_3_) δ ppm: 11.7, 14.6 (2CH_3_), 18.8 (CH_2_), 59.7 (CH_2_O), 103.6 (C-5), 111.3 (C-3), 112.7 (C-6), 115.8 (2C, *J*_C-F_ = 21.8 Hz, C-2′, C-6′), 127.9 (C-4), 128.1, 132.5 (C-4a, C-7), 128.7 (d, 2C, *J*_C-F_ = 8.4 Hz, C-3′, C-5′), 132.9 (d, *J*_C-F_ = 2.8 Hz, C-1′), 149.6 (C-2), 162.2 (d, *J*_C-F_ = 247.7 Hz, C-4′), 164.5 (COO).

Ethyl 2-(4-chlorophenyl)-7-ethylpyrrolo[1,2-*b*]pyridazine-5-carboxylate (**5f**). The compound was purified by crystallization from ethanol as fluorescent yellow crystals with mp 102–104 °C; Yield 41%. Anal. Calcd. for C_18_H_17_ClN_2_O_2_ (328.79 g/mol): C, 65.75; H, 5.21; N, 8.52. Found C, 65.57; H, 5.50; N, 8.79. IR (ATR solid, cm^−1^): 1676 (ν_C=O_); 3060 (ν_CH_). ^1^H-NMR (300 MHz, CDCl_3_) δ ppm: 1.42, 1.43 (2t, 6H, *J* = 7.1, 7.6 Hz, 2CH_3_), 3.07 (qd, 2H, *J* = 7.1, 0.9 Hz, CH_2_), 4.38 (q, 2H, *J* = 7.1 Hz, CH_2_O), 7.15 (t, 1H, *J* = 0.9 Hz, H-6), 7.24 (d, 1H, *J* = 9.5 Hz, H-3), 7.47 (d, 2H, *J* = 8.6 Hz, H-3′, H-5′), 7.94 (d, 2H, *J* = 8.6 Hz, H-2′, H-6′), 8.49 (d, 1H, *J* = 9.4 Hz, H-4). ^13^C-NMR (75 MHz, CDCl_3_) δ ppm: 11.6, 14.6 (2CH_3_), 18.8 (CH_2_), 59.8 (CH_2_O), 103.5 (C-5), 111.1 (C-3), 112.7 (C-6), 127.9 (C-4), 128.0 (C-2′, C-6′), 128.1, 132.9 (C-4a, C-7), 129.1 (C-3′, C-5′), 134.6 (C-1′), 135.8 (C-4′), 149.3 (C-2), 164.5 (COO).

### 3.2. Toxicity Evaluation

#### 3.2.1. Phytotoxicity Evaluation

Phytobiological testing (*Triticum* test) was performed by the Constantinescu method which consists in determining the maximum active dilution of a compound. Depending on the duration of action, it can influence root elongation and the cryokinetic film. It used wheat (*Triticum vulgare* Mill., Gramineae) embryonic roots as biological material because they proved to be sensitive both to the action of plant extracts [62] due to their active principles [63] and to the action of synthetic compounds [29]. Embryonic wheat roots were obtained by germinating homogeneous wheat caryopses in Linhart pots. Then the caryopses with a main root length of 1 cm were placed in the test solutions. For the determination of the action of each compound, five dilutions in the concentration range of 10–1000 µM were obtained and 11 caryopses were placed in them. Petri dishes containing 15 mL of diluted solution were kept under constant conditions of temperature (25 °C), and humidity (60%), in the absence of light. The main root was measured for three days, every 24 h considering this period to be the most active in terms of root elongation. The results were expressed in comparison with a 1% DMSO control.

Microscopic examination of *Triticum vulgare* root tips followed the mitotic film changes induced by the tested compounds. Observations were made after 24 h of contact of the caryopses with the test solutions. The study was made in comparison with DMSO control maintained under the same conditions as the samples. To obtain microscopic preparations, sectioning of wheat embryonic roots was carried out at about 5 mm from the tip and stained with dilute acetic acid stain according to the La Cour procedure [49]. The examination was conducted on a Euromex oxion series 110–240 V/50–60 Hz microscope with digital camera CEMEX 5 DC 5000 C and 40× and 100× lenses with cedar oil immersion (Sigma-Aldrich St. Louis, MO, USA).

#### 3.2.2. Animal Toxicity Assay

##### *Artemia franciscana* Toxicity Assay

The toxicity of the newly synthesized compounds was investigated by the *Artemia* test because the larvae of these primitive aquatic arthropods present in salt lakes are sensitive to a wide variety of compounds [29,64].

The biological material was commercially sourced (S.K. Trading, Thailand, which repackaged them from Ocean Star International, London, UK) and the artificial marine solution was obtained by dissolving CoralMarine Grotech sea salt in water. The necessary hatching conditions (temperature of 25 °C and continuous oxygenation) were ensured for 48 h. Dilutions in the range of 60–1000 µM were obtained from stock solutions of the new compounds. These were placed in 24-well plates in triplicate. Ten to twenty nauplii were transferred into the respective wells. After incubation for 24 h and 48 h, respectively, live and dead nauplii were counted from each well. The negative control was marine solution with 1% DMSO.

##### *Daphnia magna* Toxicity Assay

Young organisms of *Daphnia magna* were selected according to their size from a parthenogenetic culture maintained in an artificial medium for 24 h before the bioassay. The determination was conducted in tissue culture plates with 12 wells (Greiner Bio-One, Monroe, NC, USA), with 10 organisms in each well, and a final volume of 3 mL per sample [65,66]. As a negative control, DMSO was used at a concentration of 1%. All compounds were tested at six different concentration levels, ranging from 12.5 to 500 μg/mL. All assessments were performed in duplicate. Lethality was observed at both 24 h and 48 h, and the LC_50_ values were determined for each compound using the least square fit method. The LC_50_ and the 95% confidence interval (CI95%) for LC_50_ were also calculated using GraphPad Prism v 5.1 software, employing the same method.

### 3.3. Prediction of the Molecular Mechanism of Action

The newly synthesized compounds and some theoretical derivatives of their main scaffold were transformed as SMILES codes and inputted into the web application PASS, version 2.0. This algorithm uses an array of fragment types of descriptors to evaluate the potential interactions of the input compound with a large number of biologically relevant targets. The returned results consist of a list of activities and the probabilities of the compound to be active (Pa) and the probability to be inactive (Pi) [67].

### 3.4. Statistical Analyses

Statistical analyses were performed using the computing and programming environment R, R, v. 4.2.1 [68], under Rstudio, v. 2022.07.2+576 “Spotted Wakerobin” for Windows (RStudio, PBC, Boston, 2022), both for the Triticum and Artemia tests. It used a parametric mixed-effects model (R package “lme4”) [69] and a robust mixed-effects model (R package “robustlmm”) [70], in which root length measurements (Triticum) from all three days were used as the dependent variable, whereas compound, concentration and time were treated as fixed effects, with time (day of measurement) also treated as a random effect. Assessing statistical significance for mixed-effects statistical models is controversial and complex, but as a pragmatic way of working, we have estimated *p*-values using the Kenward–Rogers approximation (R package “sjPlot”) [71]. A variety of R base functions and R packages were used to diagnose the regression models (“car” [72], “MASS” [73], and “gvlma” [74]). Mixed boxplot–dotplot plots were generated using the “ggplot2” package [75].

The “drc” R package [76] was used to estimate IC_50_ values for the Triticum bioassay by nonlinear modeling. For Triticum, the relationship between root length and concentration was modeled using Weibull functions with 2, 3, or 4 parameters, depending on the data distribution, the models being selected from a range of several models with different functions and parameters, based on the Akaike’s information criterion (AIC).

### 3.5. Cell Cytotoxicity

#### 3.5.1. Cell Cultures and Treatments

For the evaluation of the in vitro potential anti-proliferative effects of treatments with the new compounds under study against solid tumor-derived cells, several compound-mediated cytotoxicity assays were performed on three adherent tumor standardized cell lines, and normal human endothelial cells, used as control: the MCF-7 human breast adenocarcinoma and SK-OV-3 human ovary adenocarcinoma cell lines were provided from European Collection of Authenticated Cell Cultures (ECACC), while LoVo, human colorectal adenocarcinoma cell line, and HUVEC human umbilical vein endothelial cells were purchased from American Type Culture Collection (ATCC) [49].

Adherent cells were cultivated in DMEM/F12 medium added with 2 mM L-glutamine and 10% fetal bovine serum, 100 units/mL penicillin, 100 µg/mL streptomycin (Sigma Aldrich, St. Louis, MO, USA) and incubated at 37 °C in 5% CO_2_ humidified atmosphere. The stock solutions for cell treatments were prepared by dissolving the synthesized compounds in a minimum amount of DMSO. Working dilutions were prepared from the stock solutions in culture medium before each treatment assay. After 24 h, when cell cultures achieved around 70% confluence, treatments were applied for various periods of time with different concentrations of synthesized compounds or oncolytic drugs, used as positive controls.

#### 3.5.2. MTS Cytotoxicity Assay

The cytotoxic activity of the new synthetized compounds was compared to that induced by several drugs that are commonly used for oncological treatments: cisplatin (Cis-Pt), 5-fluorouracil (5-FU), or doxorubicin (DOX), used as positive controls throughout our experiments. Therefore, cell cultures treated with the new compounds or oncolytic drugs for 24 h or 48 h were further subjected to a colorimetric cell viability method, the MTS assay. The absorbance of probes was read, data were calculated, and percentages of cell viability were assessed.

The evaluation of the compound-induced cytotoxicity was made using the CellTiter 96 Aqueous One Solution Cell Proliferation Assay (Promega, Madison, WI, USA), a reagent that contains both MTS (3-(4,5-dimethylthiazol-2-yl)-5-(3-carboxymethoxyphenyl)-2-(4-sulfophenyl)-2*H*-tetrazolium, inner salt) and PES (phenazine methosulfate, a cationic dye with high chemical stability that binds to MTS and forms a stable solution). The assay is based on the ability of the metabolically active cells to reduce the yellow tetrazolium salt MTS to a compound that is soluble in culture medium, the colored formazan, followed by the spectrophotometric evaluation of its concentration.

Briefly, 1.5 × 10^4^ cancer or normal cells were cultured in 100 µL/well for 24 h. After the culture supernatants were discarded, cells were treated for additional 24 h or 48 h with increasing concentrations of new compounds or oncolytic drugs. Following the specific treatments, in each well a volume of 20 µL of coloring mixture reagent of MTS and PES was added, and then the plates were incubated with mild agitation every 20 min for 4 h at 37 °C. The color developed during incubation was spectrophotometrically quantified at λ = 492 nm using a Dynex ELISA reader (DYNEX Technologies—MRS, Chantilly, VA, USA) [51,52,55].

Data were expressed as percentages of cell viability of the treated cells, and were calculated and compared to the untreated cells (considered 100% viable), using the formula:

Cell viability (%) = (T − B)/(U − B) × 100

where T = absorbance of treated cells, U = absorbance of untreated cells, and B = absorbance of culture medium (blank), for λ = 492 nm.

The cell viability data were expressed as the mean values ± standard deviations (SD) of three different experiments (n = 3). In addition, the MTS assay was performed in the same experimental conditions for the evaluation of DMSO potential cytotoxicity, using serial dilutions of the reagent; no cell cytotoxicity was observed in DMSO concentrations lower than 1% [49,52]. Moreover, a parallel experiment was performed in the absence of cells, with all the concentrations of the compounds being tested for their potential interference with MTS reagents; then, their absorbance values were extracted during calculations.

#### 3.5.3. Statistical Analysis

All cytotoxicity assays were performed in triplicate (n = 3) and expressed as mean values ± standard deviations (SD). Statistical analyses were carried out using one-way analysis of variance (ANOVA) test; *p* values < 0.05 were considered statistically significant.

## 4. Conclusions

New pyrrolo[1,2-*b*]pyridazines were synthesized, with moderate yields, by 3 + 2 cycloaddition reaction between mesoionic oxazolo-pyridazinones and terminal activated alkyne dipolarophiles and their cytotoxicity was evaluated. The obtaining of mesoionic oxazolo-pyridazinones intermediate took place in situ from the 3(2*H*)pyridazinone acids in the presence of acetic anhydride. In the first stage, 3(2*H*)pyridazinone esters were synthesized by multi-step synthesis that led to the corresponding acids by hydrolysis. The spectral analysis (IR, ^1^H-, ^13^C-NMR), X-ray diffraction, and elemental analysis confirmed the structures of the synthesized compounds. The toxicity studies on *Triticum aestivum* L. cells indicated that the toxicity was low for all compounds, with IC_50_ higher than 200 µM, the acids **2b** and **2a** having the lowest values. The toxicity studies on crustaceans indicated that except for **5a** and **5c** could have a toxicity effect, the newly synthetized compounds showed moderate or no toxicity on Daphnia, while on Artemia nauplii no lethality was induced. The cytotoxic effects of the compounds on three human adenocarcinoma-derived adherent cell lines (colon LoVo, ovary SK-OV-3, and breast MCF-7) and on HUVEC endothelial cells highlighted that several of these display satisfactory anticancer activities, and very low cytotoxic effects towards normal cells. The in vitro compound-mediated cytotoxicity assays demonstrated dose- and time-dependent cytotoxic activity for several newly synthesized compounds, the highest anti-tumor properties, based on the cell viability, being assessed for acid **2a** and its derivative **5a**, and for **2c** and the derivatives **5c** and **5f**, especially against colon cancer cells. Among them, compounds **5a**, **2c,** and **5f** showed the lowest IC_50_ values on the LoVo cell line. The obtained results prompted us to improve the anti-cancer properties of the most promising tested compounds, and further expand our studies on their biological activities, in order to modulate the chemo-sensitivity of tumor cells to innovative drug treatments that might overcome or reverse the chemo-resistance usually found in cancer patients after several cycles of chemotherapy.

## Figures and Tables

**Table 1:** Crystal data and details of structure refinement.

Compound	1c	2b	5a
Emp. formula	C_16_H_17_ClN_2_O_3_	C_14_H_13_FN_2_O_3_	C_34_H_32_N_4_O_4_
Fw	320.76	276.26	560.63
T (K)	160	160	160
space group	*P*2_1_/*c*	*C*c	*P*-1
*a* (Å)	8.9182(4)	5.2160(7)	8.0870(8)
*b* (Å)	12.6490(6)	28.809(4)	12.3024(11)
*c* (Å)	14.1341(6)	8.4911(12)	15.0450(12)
*α* (°)	90	90	78.328(7)
β (°)	101.940(5)	99.957(14)	84.704(7)
γ (°)	90	90	70.883(9)
*V* (Å^3^)	1559.93(12)	1256.8(3)	1384.5(2)
*Z*	4	4	2
ρ_calcd_ (g cm^−3^)	1.366	1.460	1.345
μ (mm^−1^)	0.259	0.114	0.089
Crystal size (mm)	0.20 × 0.20 × 0.15	0.20 × 0.10 × 0.04	0.25 × 0.2 × 0.1
2Θ range	4.364 to 50.038	5.632 to 56.462	3.564 to 50.052
Refls. collected	5978	4054	13,500
Indep. Refls., *R*_int_	2750, 0.0697	1917, 0.1186	4897, 0.0526
Data/rests./params.	2750/0/201	1917/2/171	4897/0/383
GOF	1.038	1.035	1.046
*R*_1,_*wR*_2_ (all data)	0.0498, 0.1245	0.0853, 0.2328	0.0447, 0.1147
CCDC no.	2,267,177	2,267,178	2,267,179

**Table 2:** IC_50_ values for compounds **2a**–**c** and **5e**.

Compound	IC_50_ (μM)	95% Confidence Interval
**2a**	213.1	129.8–296.4
**2b**	204.3	127.4–281.1
**2c**	552.7	408.7–696.8
**5e**	606.6	472.6–740.7

**Table 3:** Daphnia magna bioassay at 48 h.

Compound	LC_50_ (μM)	95% Confidence Interval (μM)
**2a**	521.3	ND
**2b**	ND *	ND
**2c**	ND *	ND
**5a**	46.12	35.52 to 59.89
**5b**	463.4	ND
**5c**	ND ** (~106.8)	ND
**5e**	675.3	ND
**5f**	427.9	205.4 to 891.7

LC_50_—50% lethal concentration; ND—not determined due to lethality values; ND *—all L% values were lower than 30%; ND **—all L% values were in range of 30 to 70%, independent of concentration.

**Table 4:** IC_50_ values for newly synthesized compounds and drug-mediated tumor cell lysis.

	LoVo		MCF-7		Sk-OV-3	
**Compound**	**24 h**	**48 h**	**24 h**	**48 h**	**24 h**	**48 h**
**2a**	≥400 µM	≥400 µM	ND	≥400 µM	≥400 µM	≥400 µM
**5a**	≥400 µM	303.78 ± 4.85	ND	ND	ND	ND
**2b**	≥400 µM	≥400 µM	ND	≥400 µM	≥400 µM	≥400 µM
**5b**	≥400 µM	≥400 µM	ND	≥400 µM	≥400 µM	≥400 µM
**5e**	≥400 µM	≥400 µM	≥400 µM	≥400 µM	≥400 µM	≥400 µM
**2c**	≥400 µM	366.84 ± 3.70	ND	ND	≥400 µM	≥400 µM
**5c**	≥400 µM	≥400 µM	ND	≥400 µM	≥400 µM	≥400 µM
**5f**	≥400 µM	353.32 ± 4.10	ND	≥400 µM	≥400 µM	≥400 µM
5-FU	76.08 ± 3.09	66.25 ± 2.66	80.63 ± 4.39	60.43 ± 5.04	NT	NT
CisPt	79.07 ± 4.75	57.01 ± 0.95	NT	NT	92.19 ± 3.45	78.23 ± 4.01
DOX	NT	NT	14.80 ± 4.00	11.60 ± 0.53	55.41 ± 2.98	12.10 ± 0.32

IC_50_ values represent the concentrations of a tested compound required to inhibit 50% of the cell growth, with respect to the control sample (in the absence of the tested compound), and they are presented as mean ± SD of three independent experiments; NT—not tested; ND—not determined due to lethality values.

## Data Availability

The data presented in this study are available upon request from the corresponding authors.

## Supplementary Materials

The following supporting information can be downloaded at: https://www.mdpi.com/article/10.3390/ijms241411642/s1.
