# Supplementary material for: Synthesis, Characterization and Cytotoxic Evaluation of New Pyrrolo[1,2-b]pyridazines Obtained via Mesoionic Oxazolo-Pyridazinones
Source: Int J Mol Sci. 2023 Jul 19;24(14):11642. doi: 10.3390/ijms241411642 (PMC10380841; doi:10.3390/ijms241411642)
Supplement: Supplementary file 1 [file ijms-24-11642-s001.zip › ijms-2489227-supplementary.pdf]

International Journal of *Molecular Sciences*

MDPI

Article

Synthesis, Characterization and Cytotoxic Evaluation of New Pyrrolo[1,2-*b*]pyridazines Obtained *via* Mesoionic Oxazolo-pyridazinones

Beatrice-Cristina Ivan [1], Stefania-Felicia Barbuceanu [1,*], Camelia Mia Hotnog [2], Octavian Tudorel Olaru [3], Adriana Iuliana Anghel [3], Robert Viorel Ancuceanu [3], Mirela Antonela Mihaila [2], Lorelei Irina Brasoveanu [2], Sergiu Shova [4,*], Constantin Draghici [5], George Mihai Nitulescu [6] and Florea Dumitrascu [5]

1. The IR spectrum

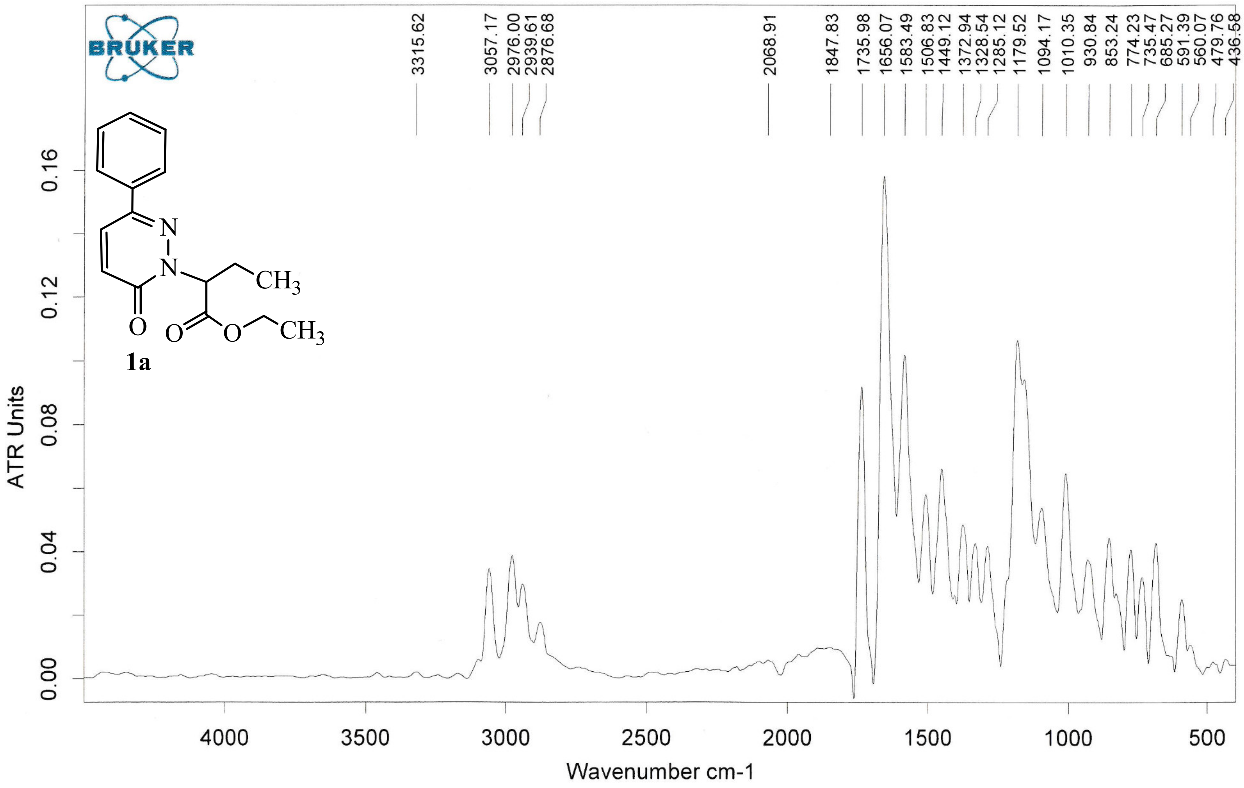

Figure 1S. The IR spectrum of ester **1a**

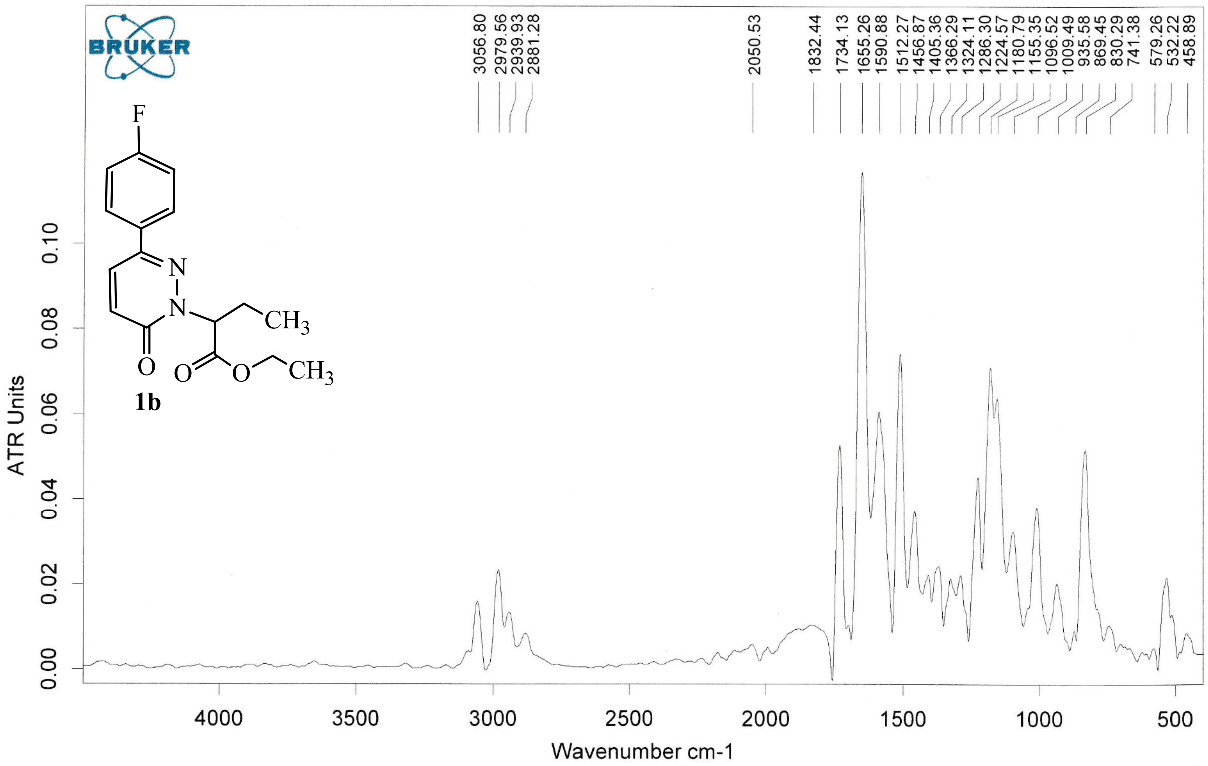

Figure 2S. The IR spectrum of ester **1b**

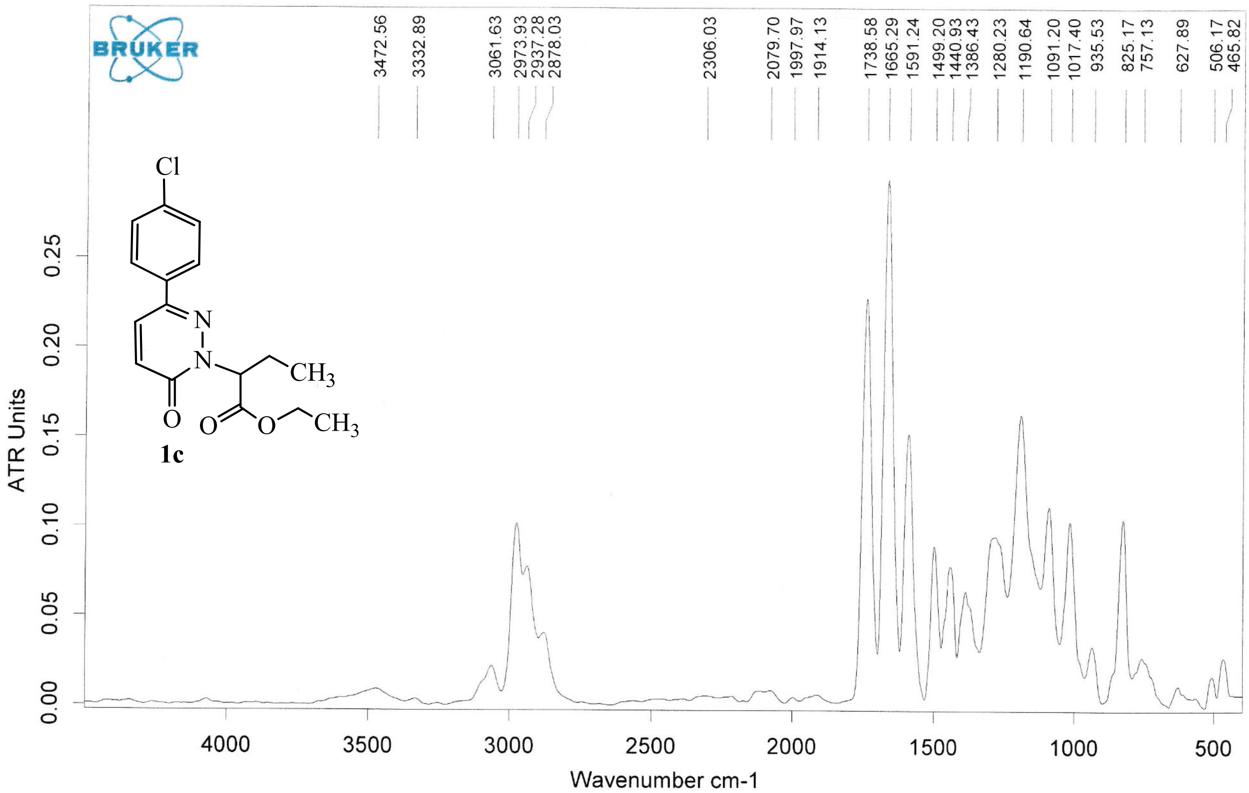

Figure 3S. The IR spectrum of ester **1c**

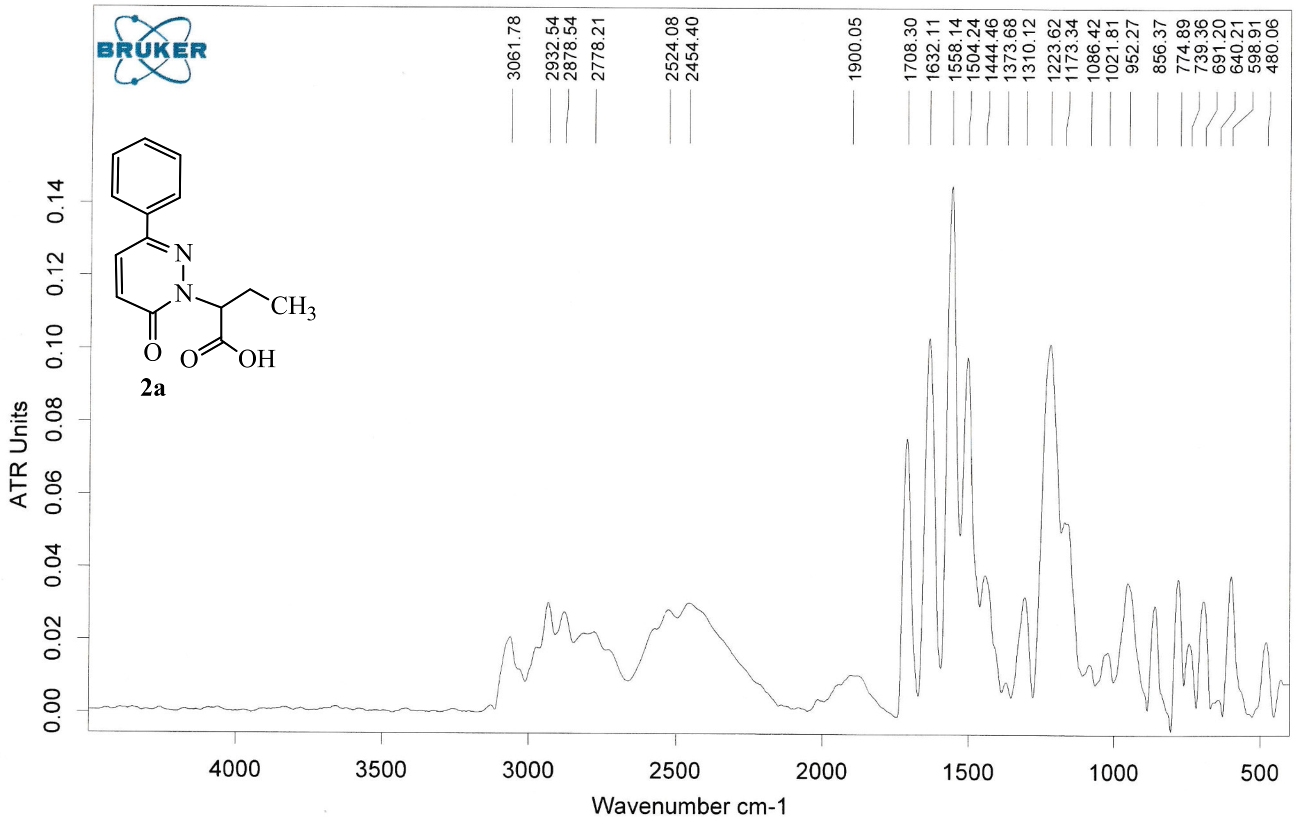

Figure 4S. The IR spectrum of acid **2a**

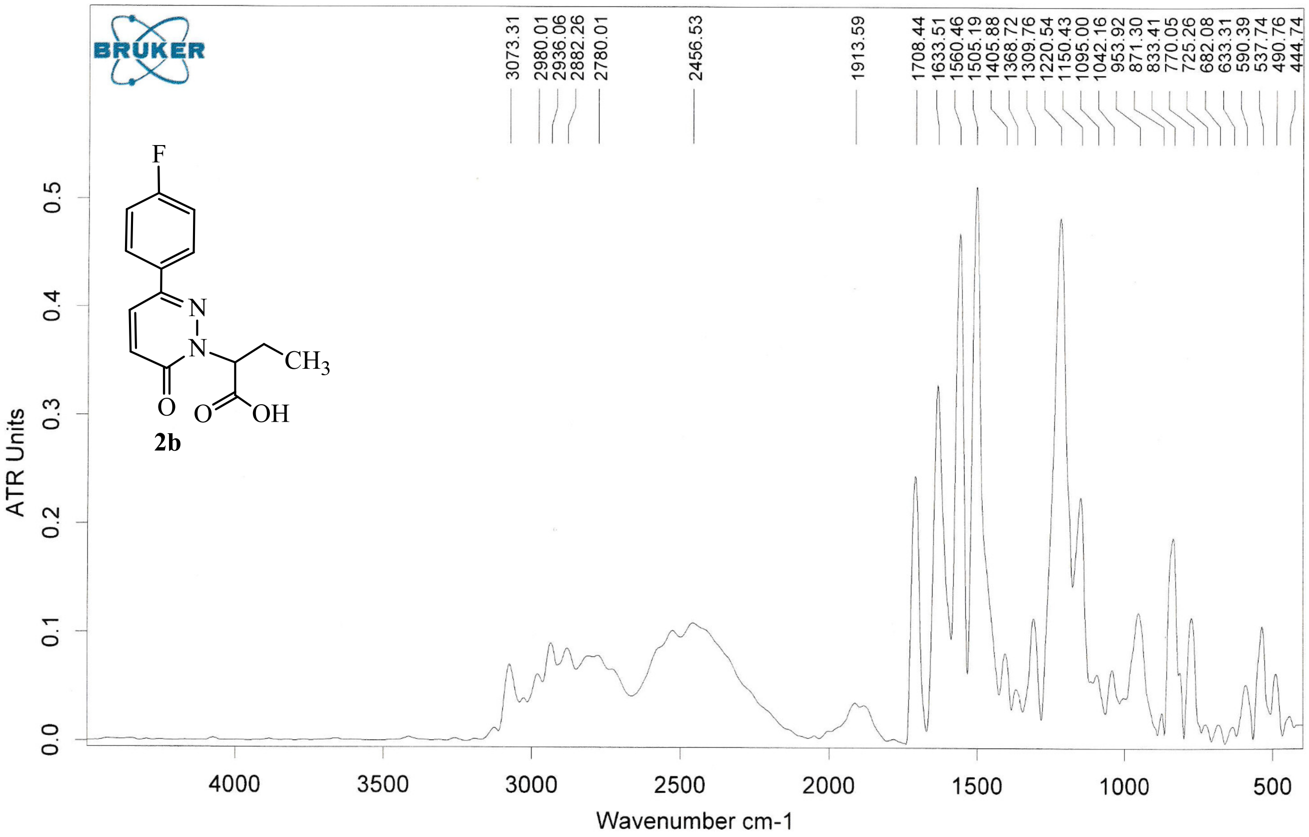

Figure 5S. The IR spectrum of acid **2b**

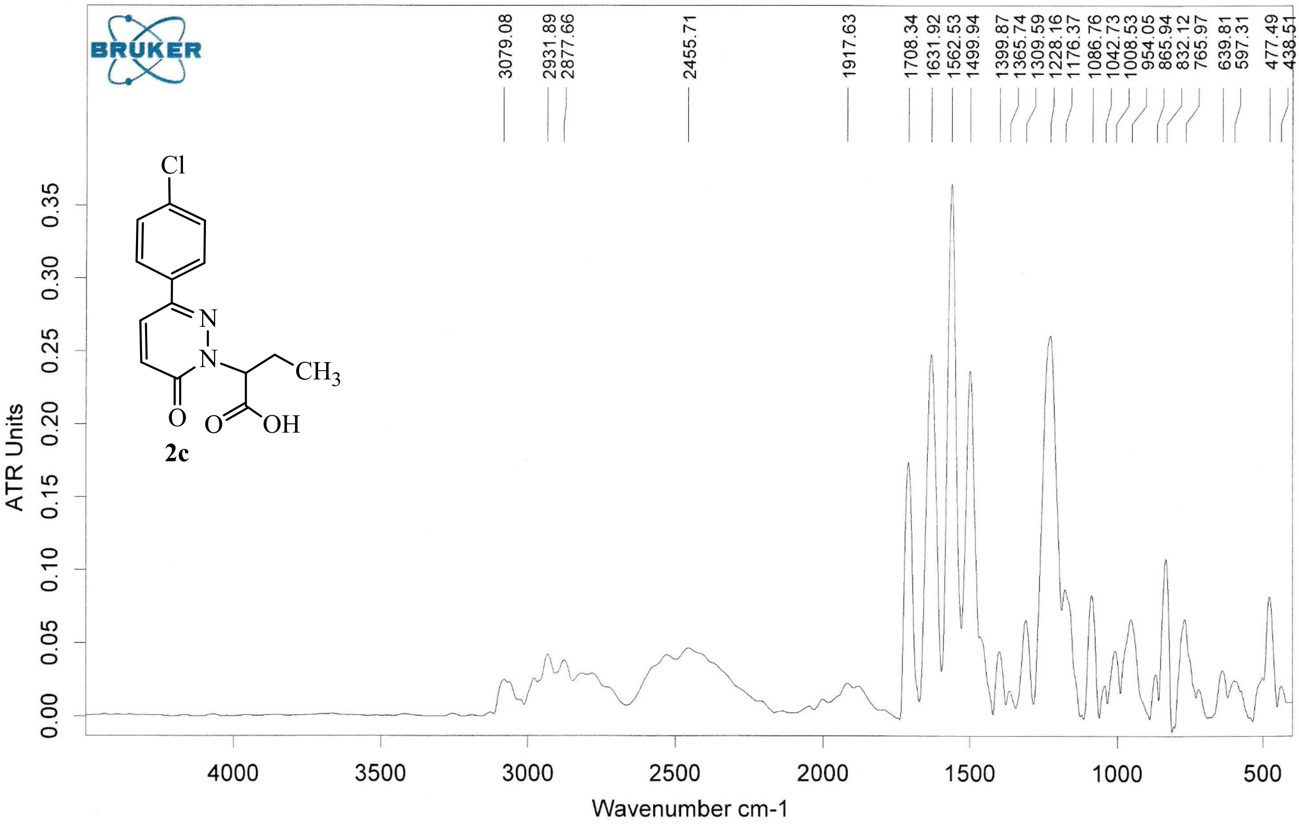

Figure 6S. The IR spectrum of acid **2c**

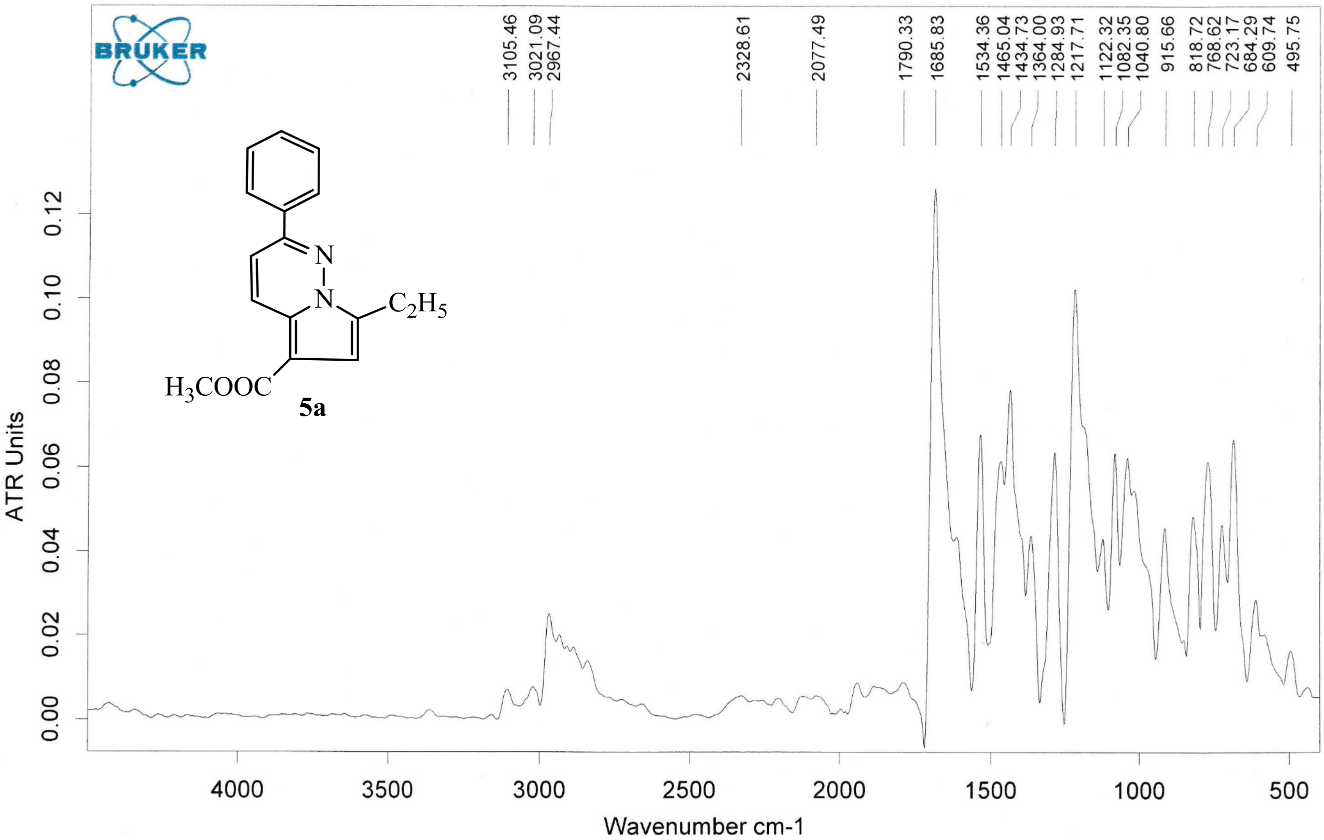

Figure 7S. The IR spectrum of pyrrolo[1,2-*b*]pyridazine **5a**

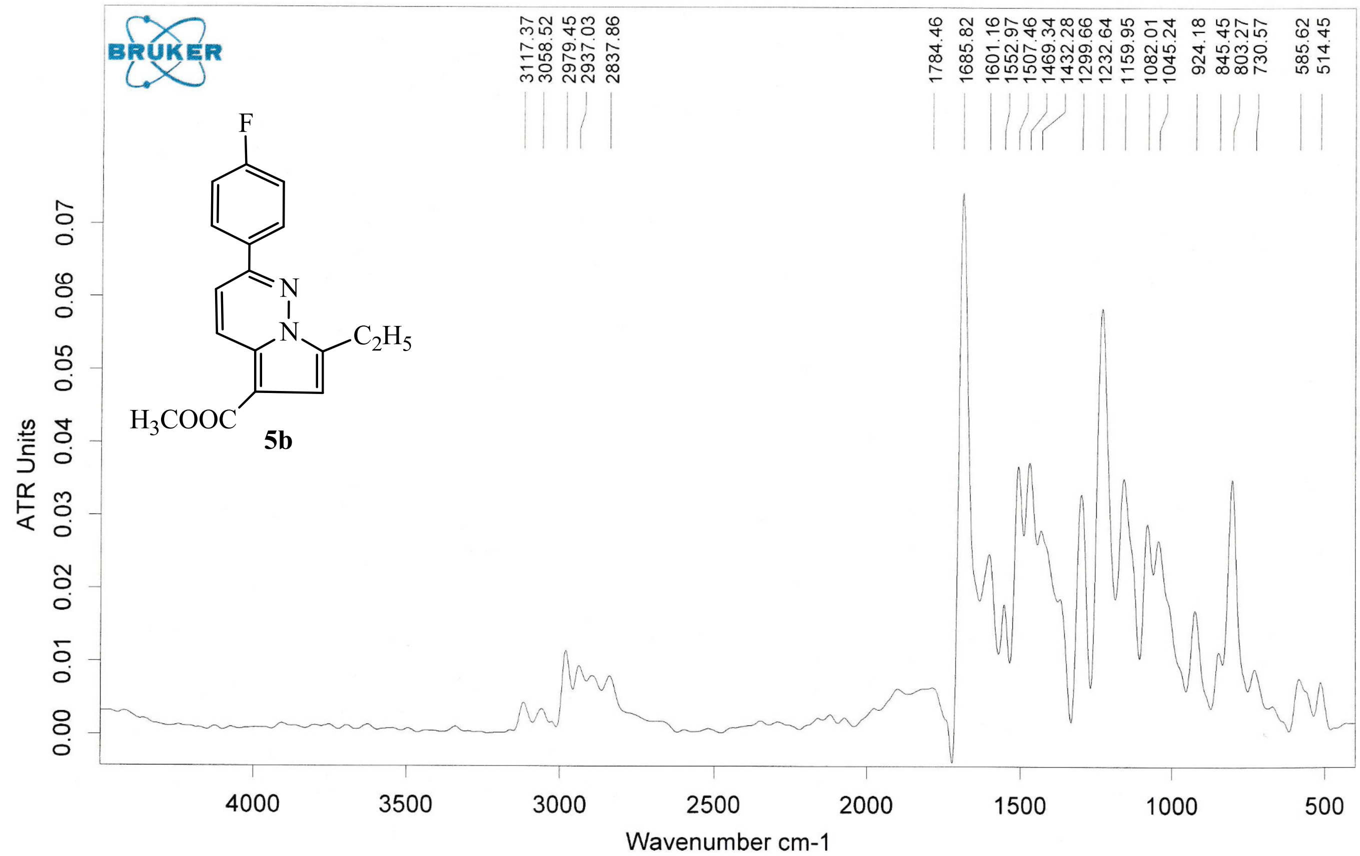

Figure 8S. The IR spectrum of pyrrolo[1,2-*b*]pyridazine **5b**

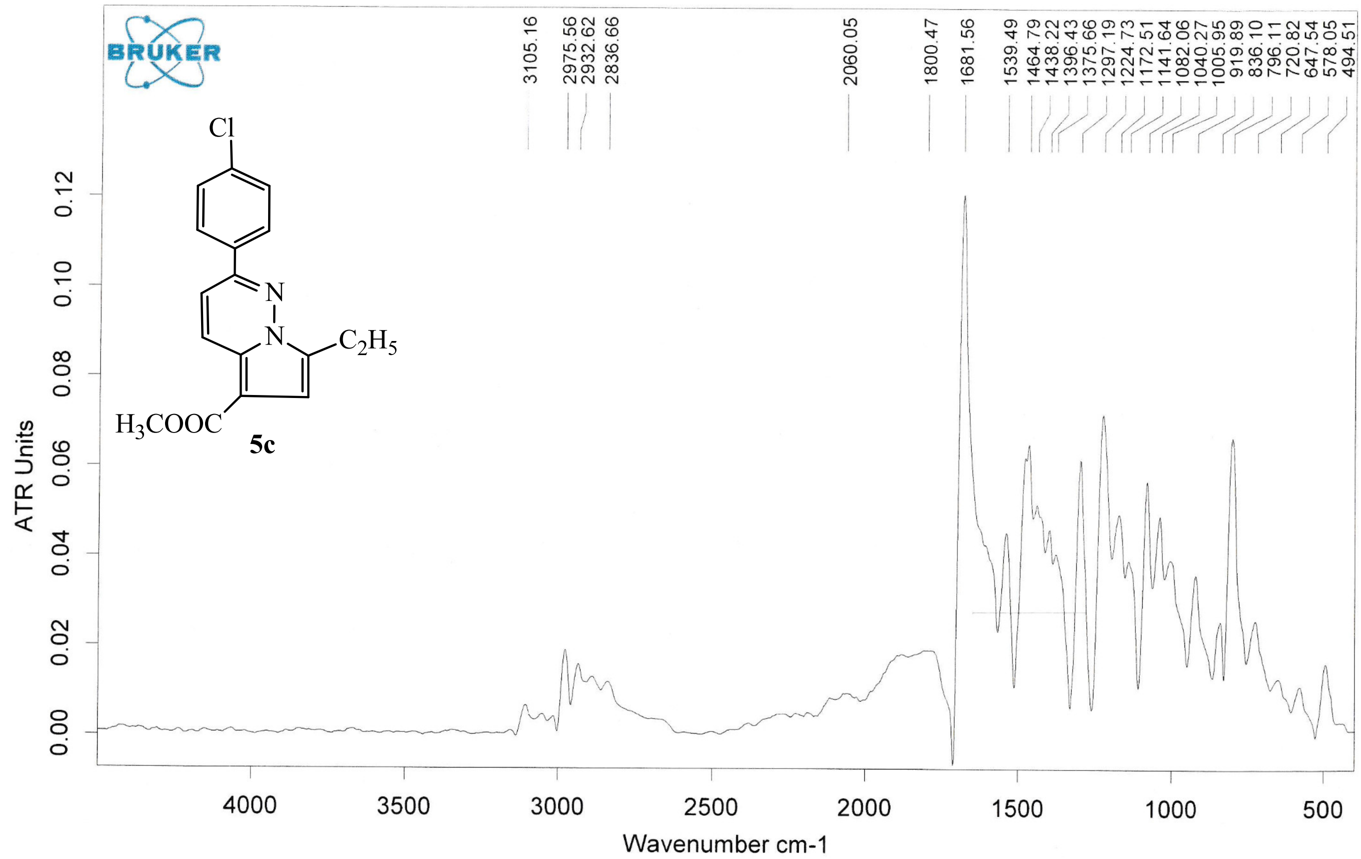

Figure 9S. The IR spectrum of pyrrolo[1,2-*b*]pyridazine **5c**

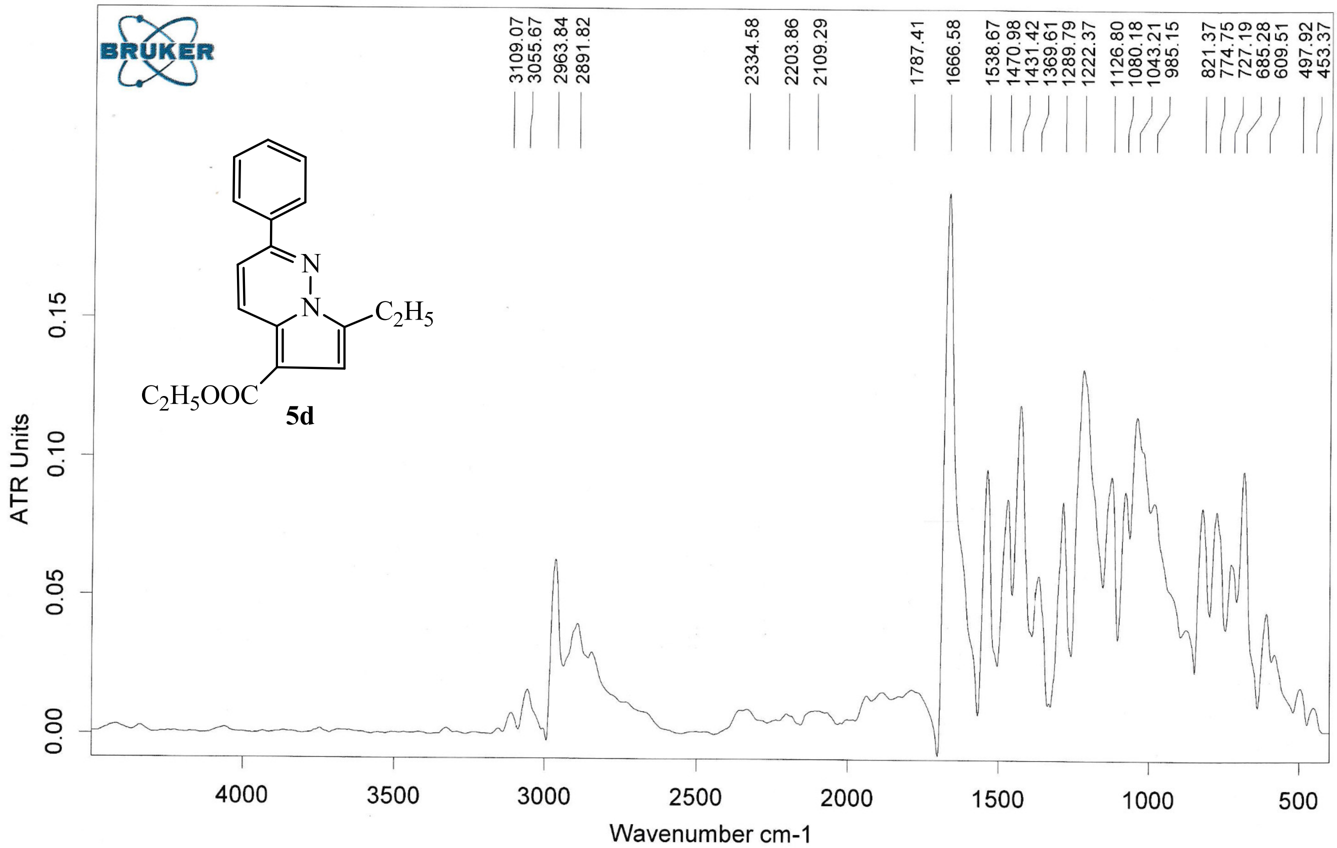

Figure 10S. The IR spectrum of pyrrolo[1,2-*b*]pyridazine **5d**

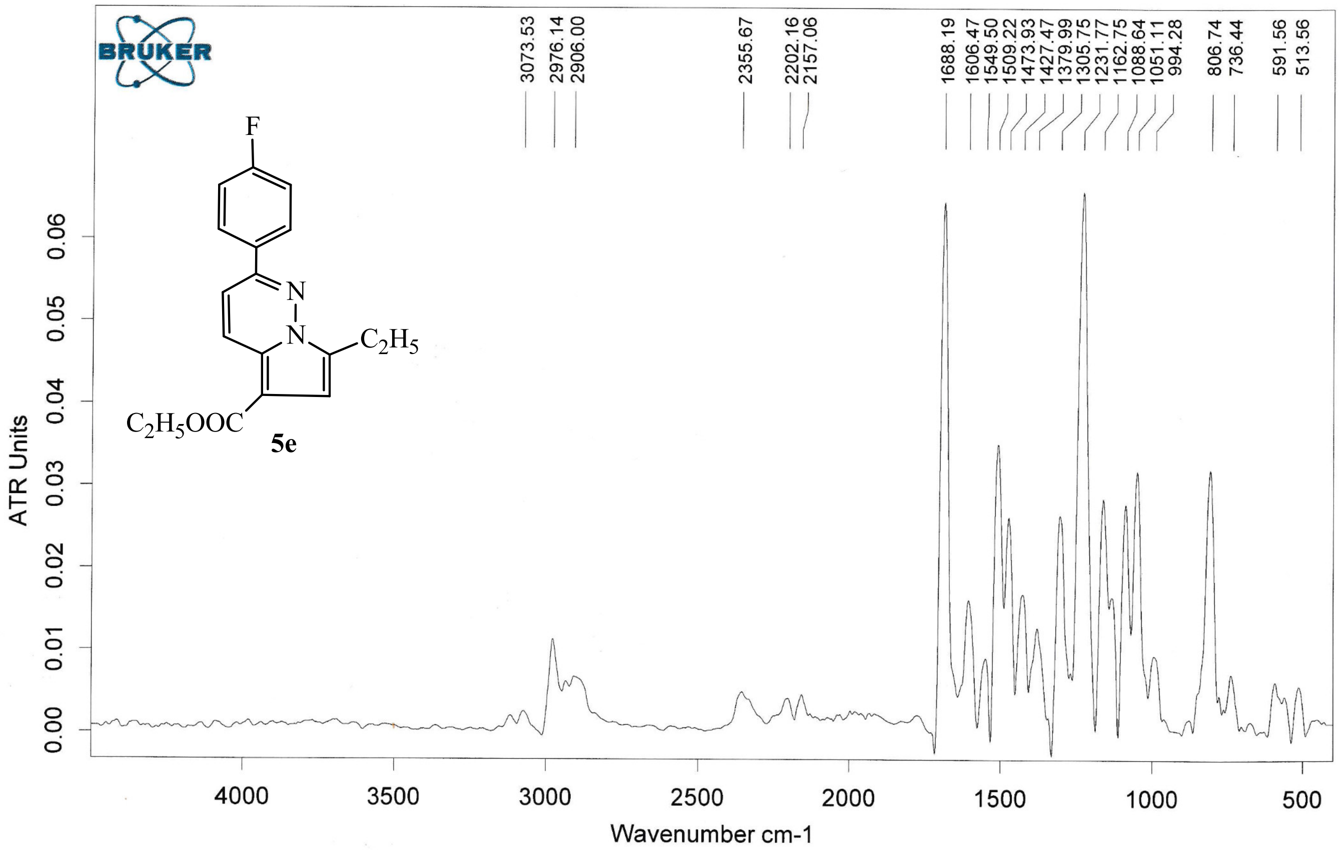

Figure 11S. The IR spectrum of pyrrolo[1,2-*b*]pyridazine **5e**

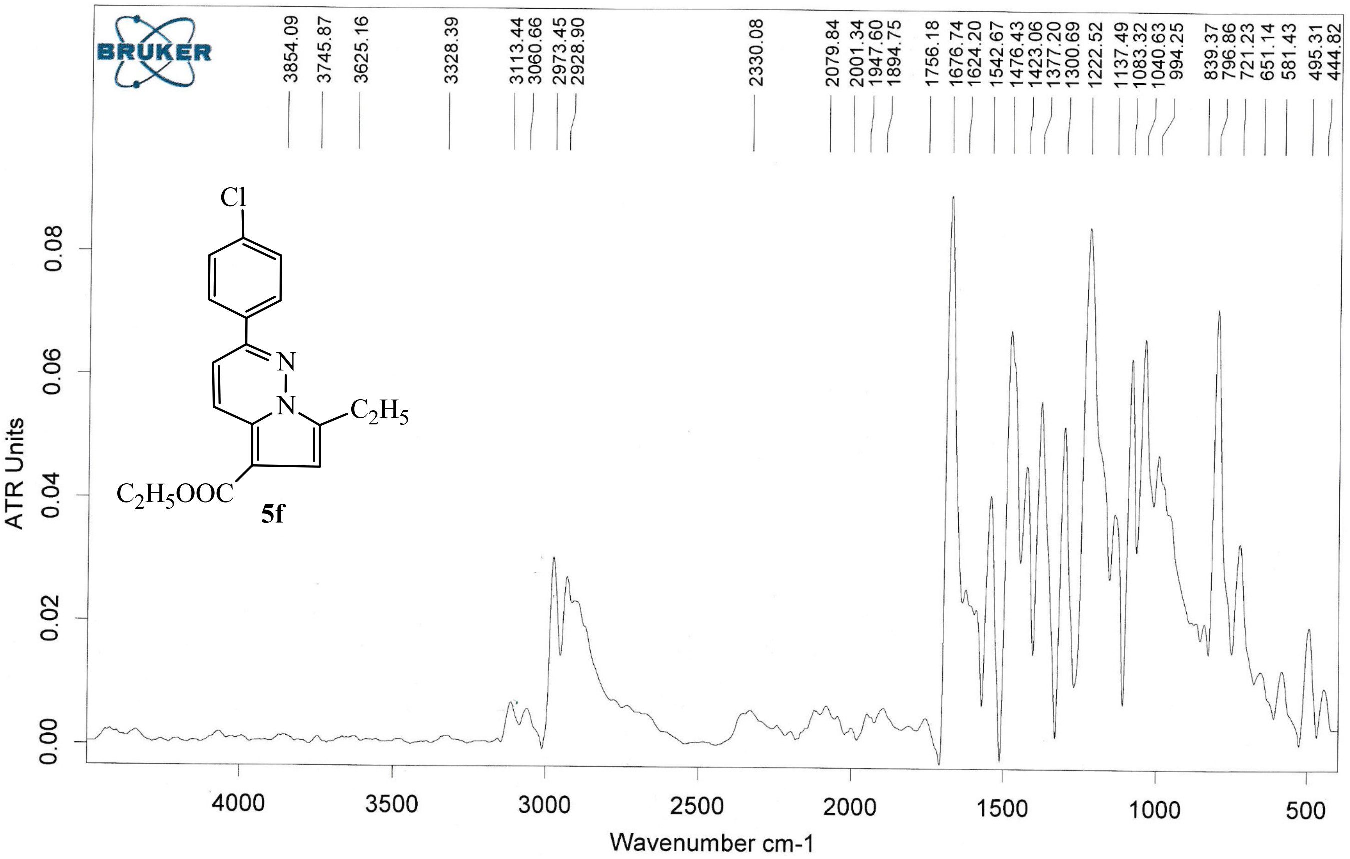

Figure 12S. The IR spectrum of pyrrolo[1,2-*b*]pyridazine **5f**

2. The ^{1}H-NMR and ^{13}C-NMR spectra

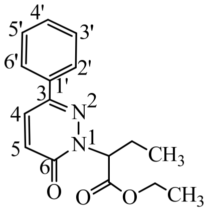

1a

Figure 13S. The ^{1}H-NMR spectrum of ester **1a**

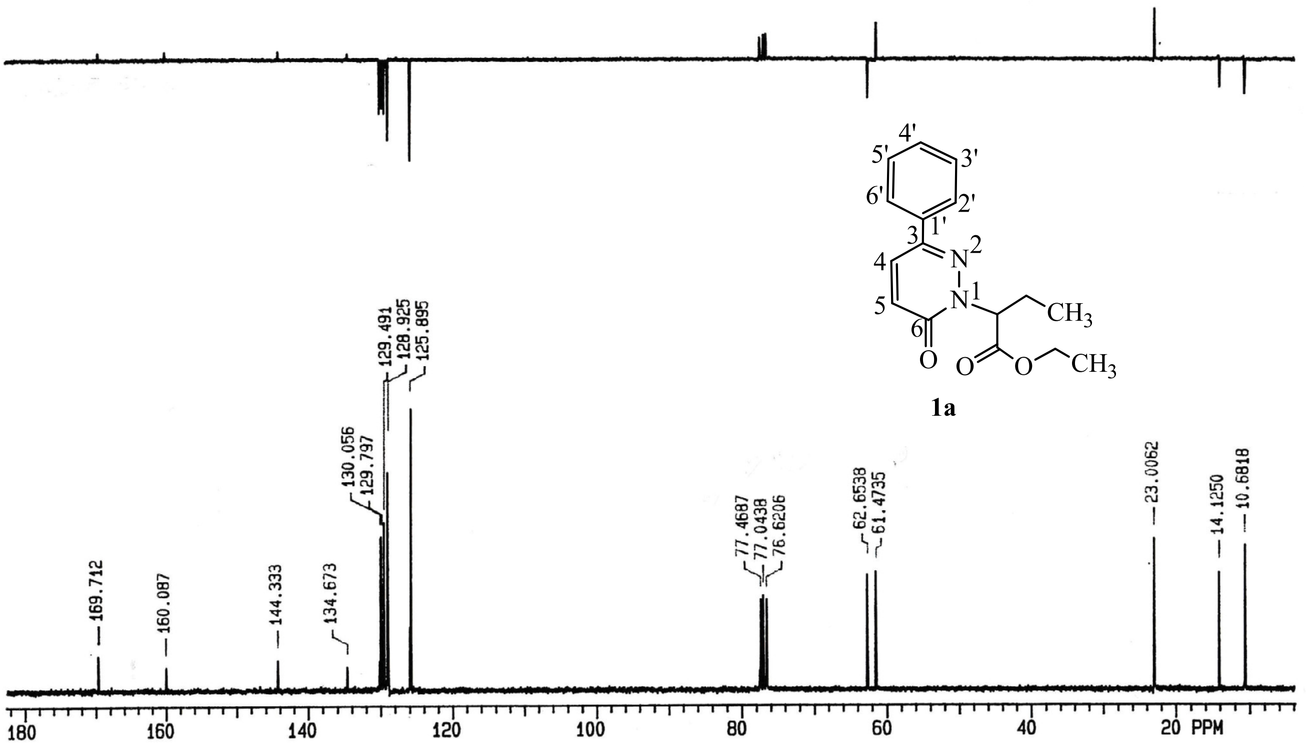

Figure 14S. The ^{13}C-NMR spectrum of ester **1a**

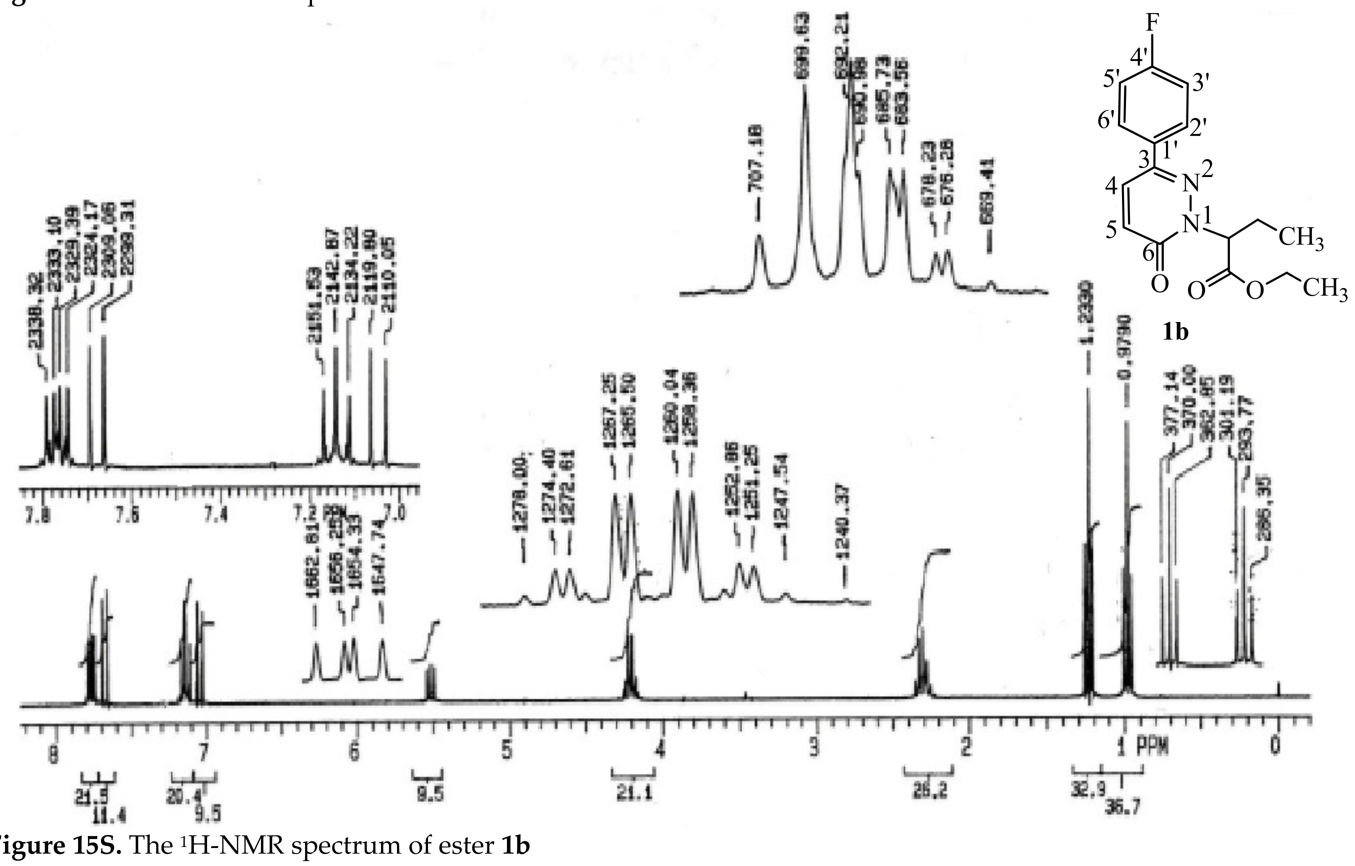

Figure 15S. The ^{1}H-NMR spectrum of ester **1b**

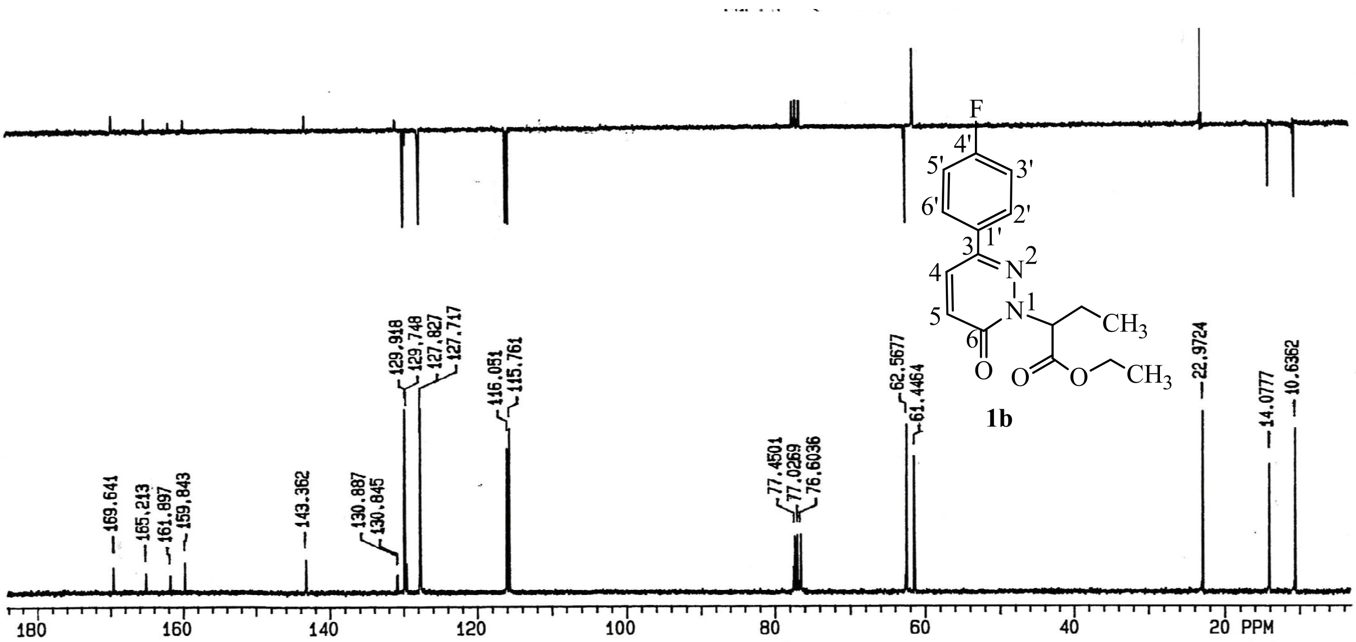

Figure 16S. The ^{13}C-NMR spectrum of ester **1b**

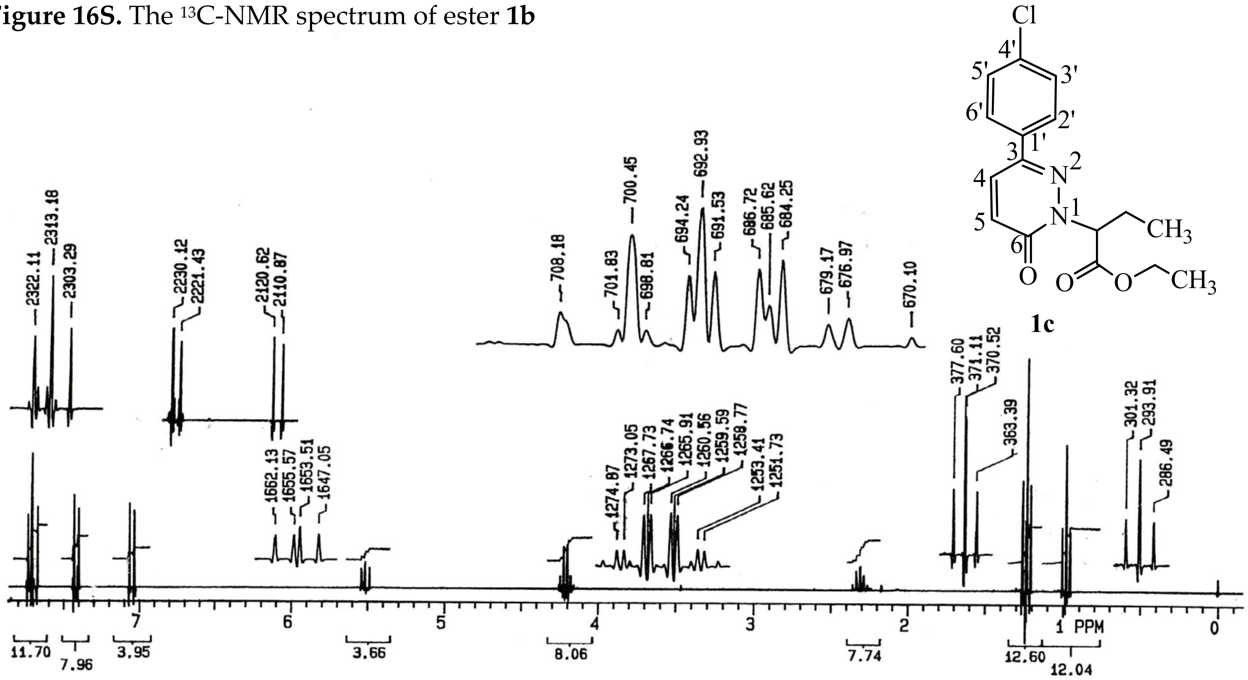

Figure 17S. The ^{1}H-NMR spectrum of ester **1c**

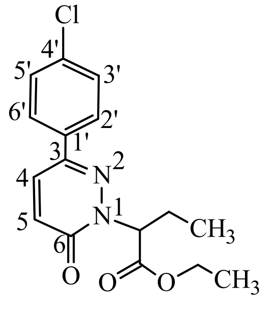

1c

Figure 18S. The ^{13}C-NMR spectrum of ester **1c**

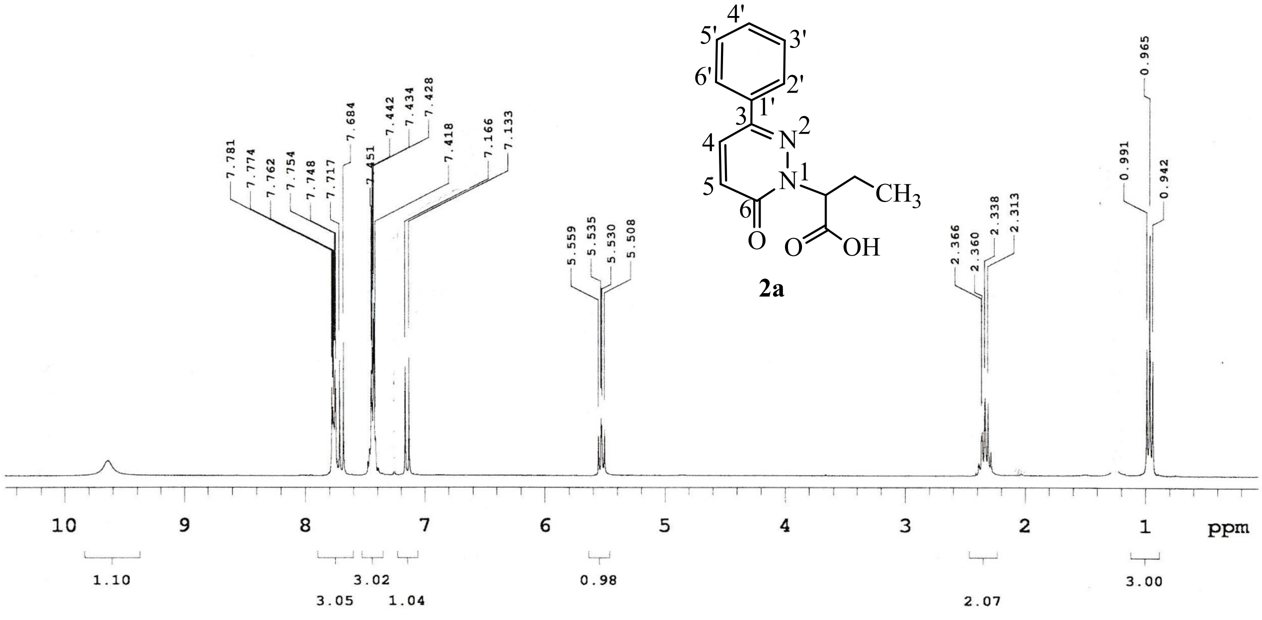

Figure 19S. The ^{1}H-NMR spectrum of acid **2a**

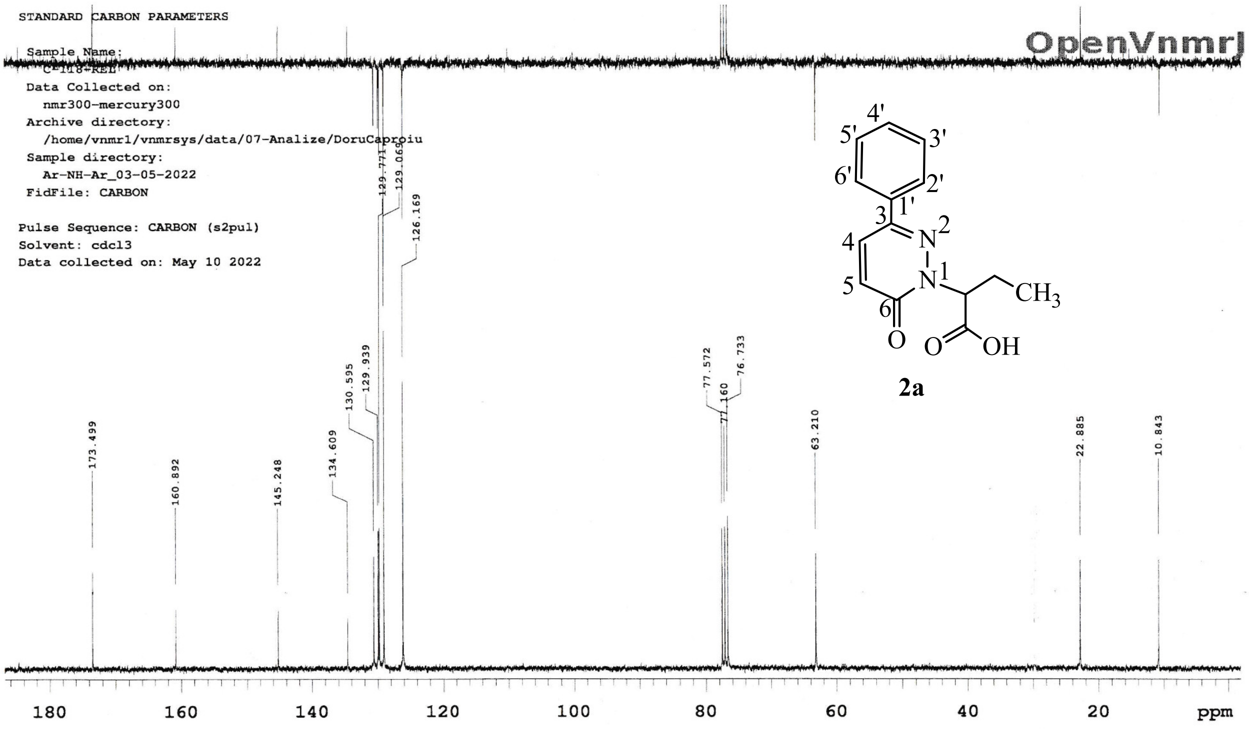

Figure 20S. The ^{13}C-NMR spectrum of acid **2a**

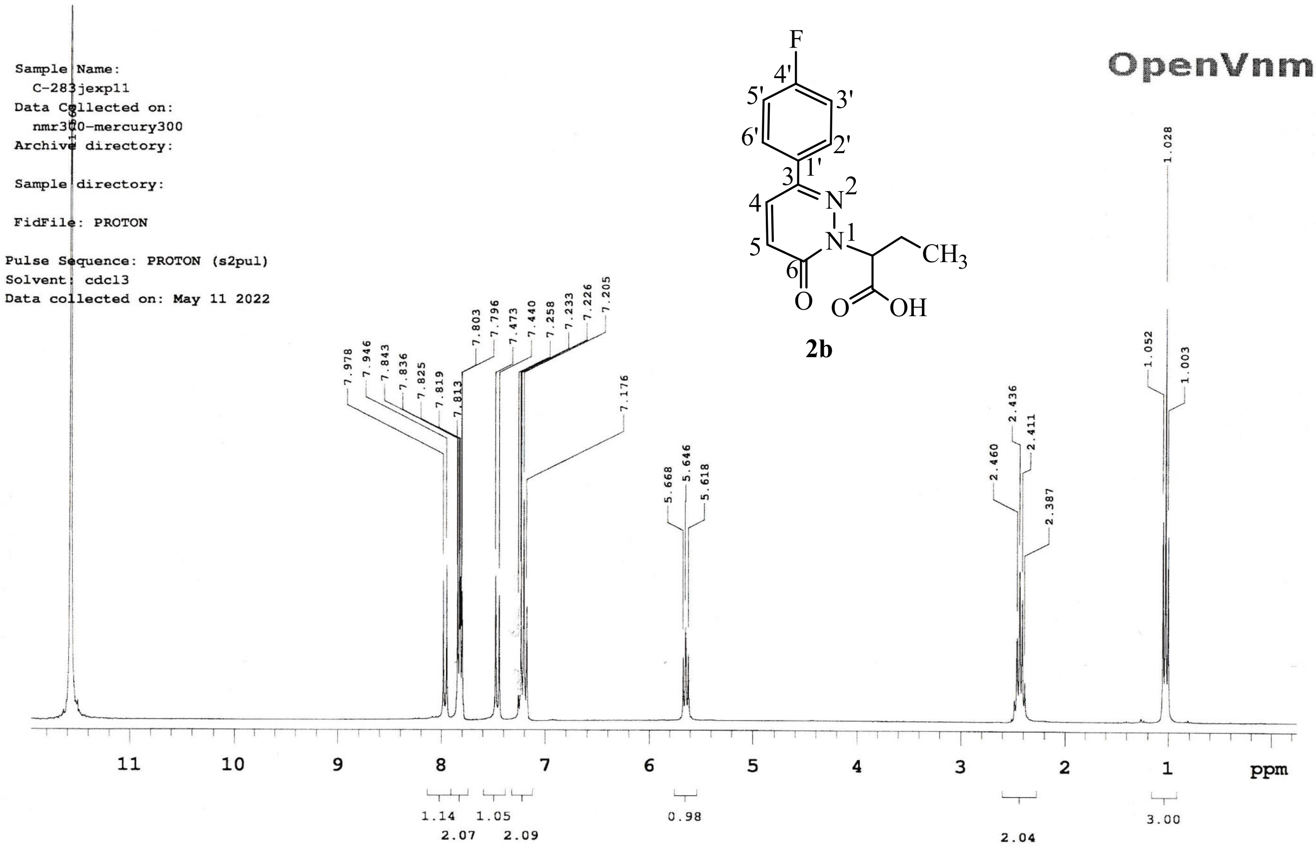

Figure 21S. The ^{1}H-NMR spectrum of acid **2b**

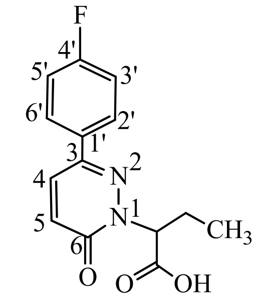

2b

Figure 22S. The ^{13}C-NMR spectrum of acid **2b**

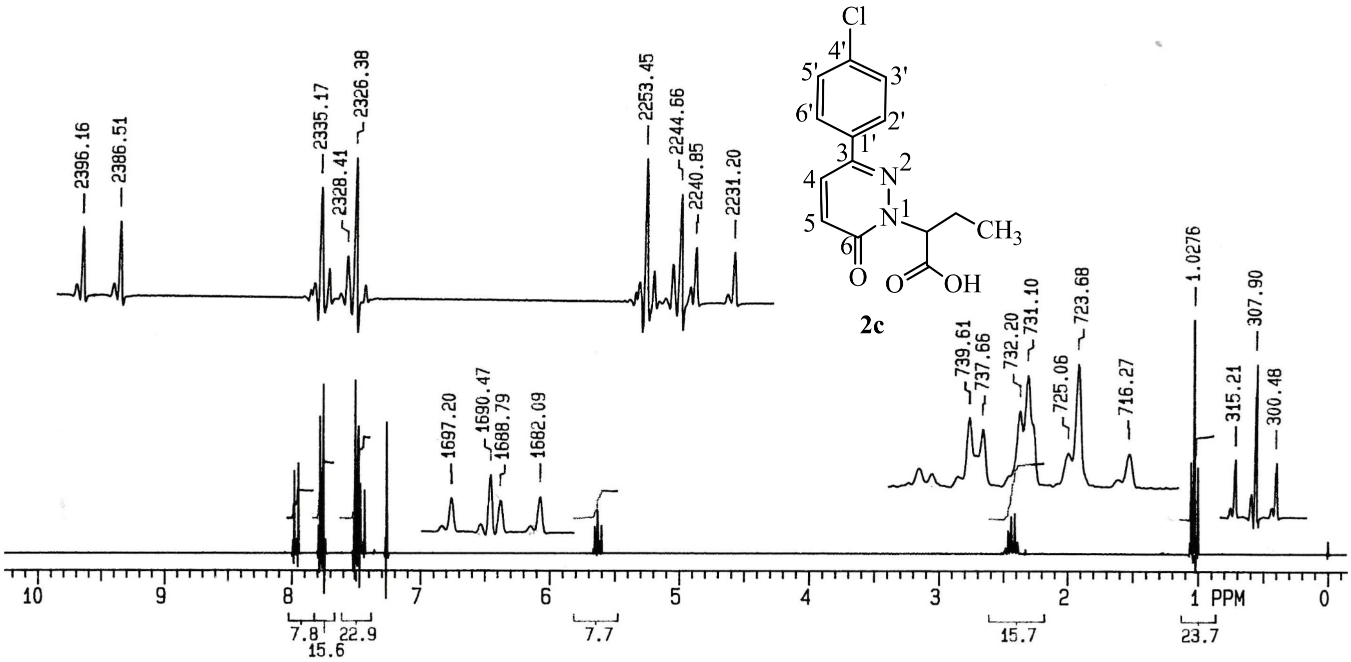

Figure 23S. The ^{1}H-NMR spectrum of acid **2c**

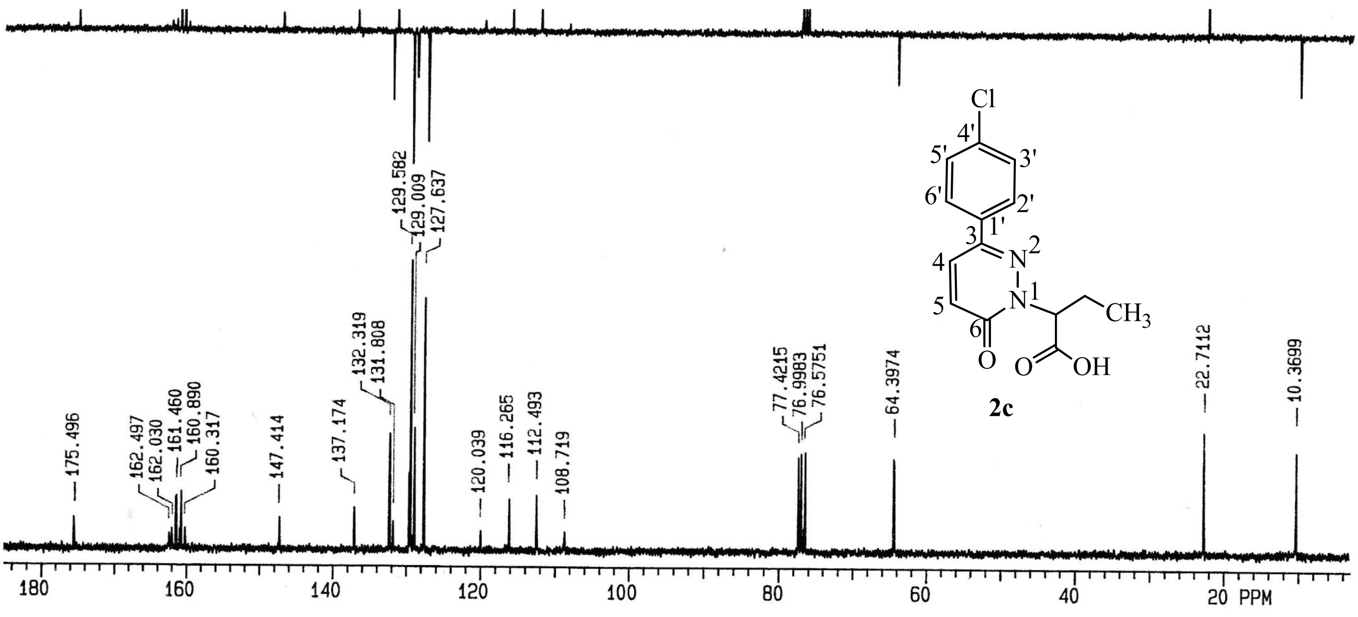

Figure 24S. The ^{13}C-NMR spectrum of acid **2c**

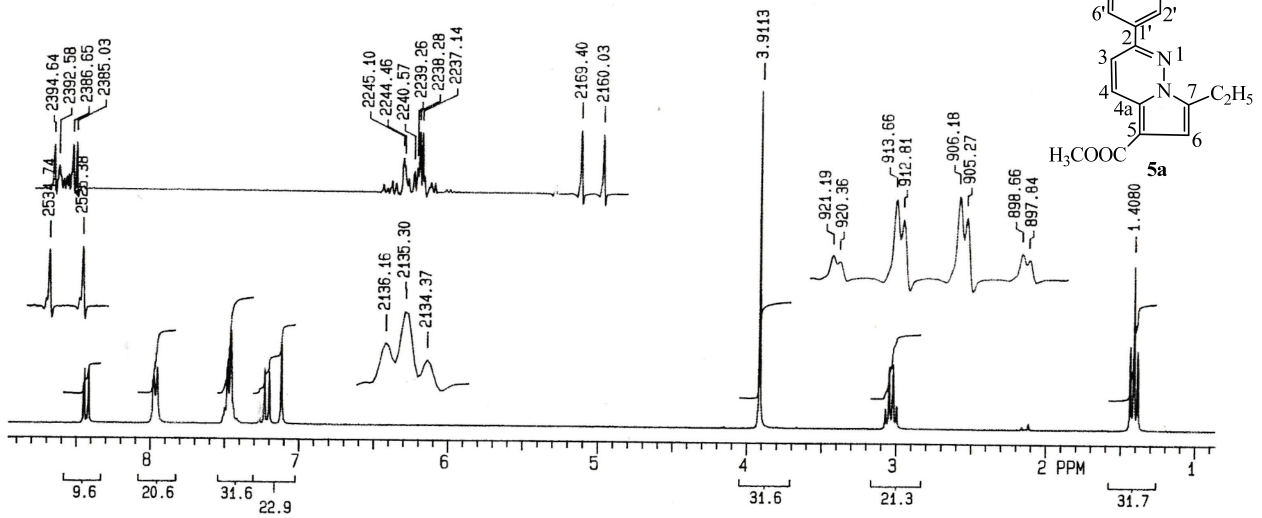

Figure 25S. The ^{1}H-NMR spectrum of **5a**

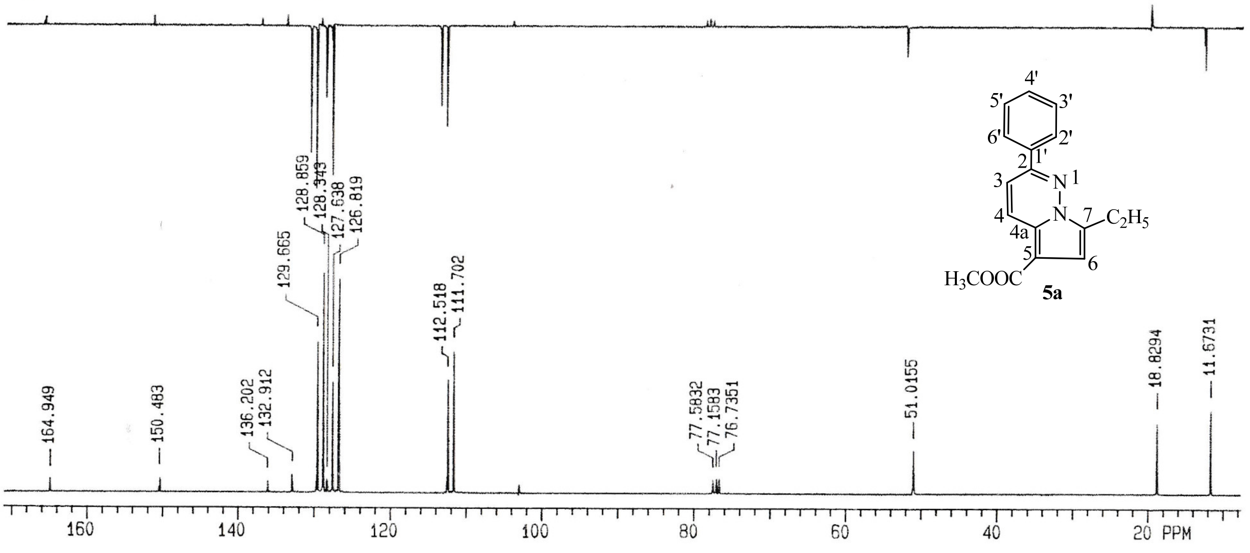

Figure 26S. The ^{13}C-NMR spectrum of **5a**

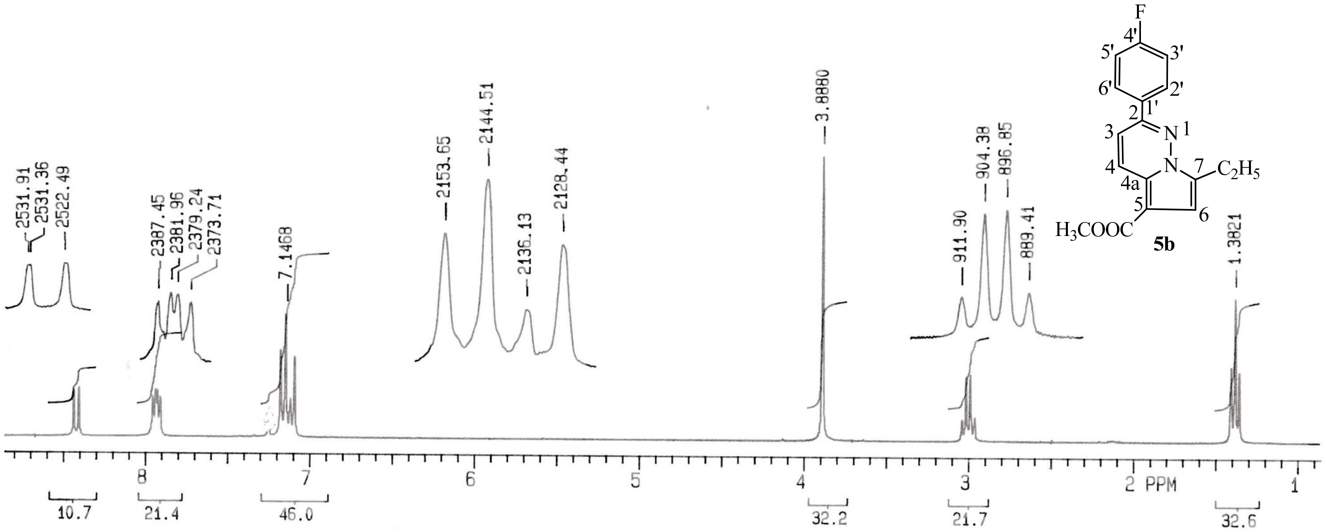

Figure 27S. The ^{1}H-NMR spectrum of **5b**

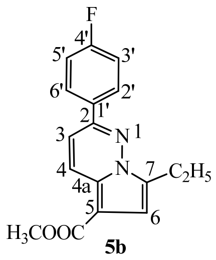

Figure 28S. The ^{13}C-NMR spectrum of **5b**

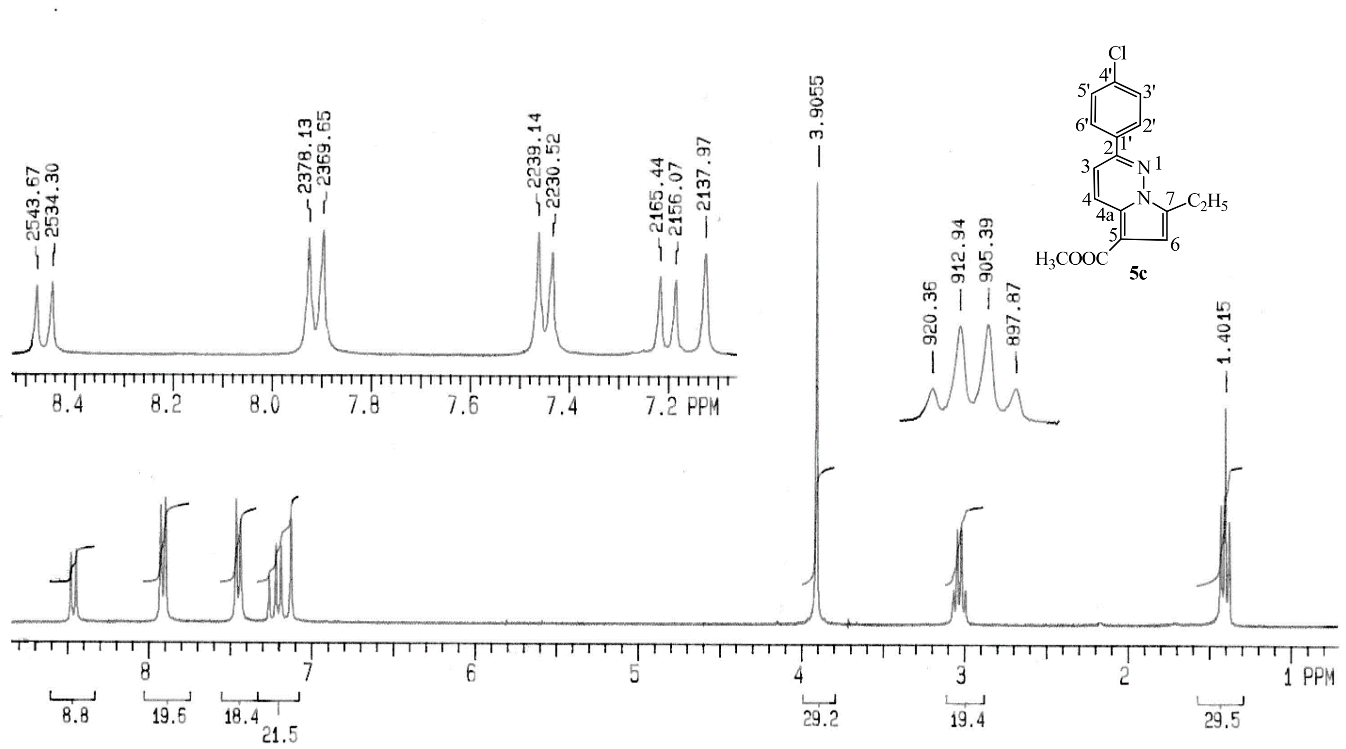

Figure 29S. The ^{1}H-NMR spectrum of **5c**

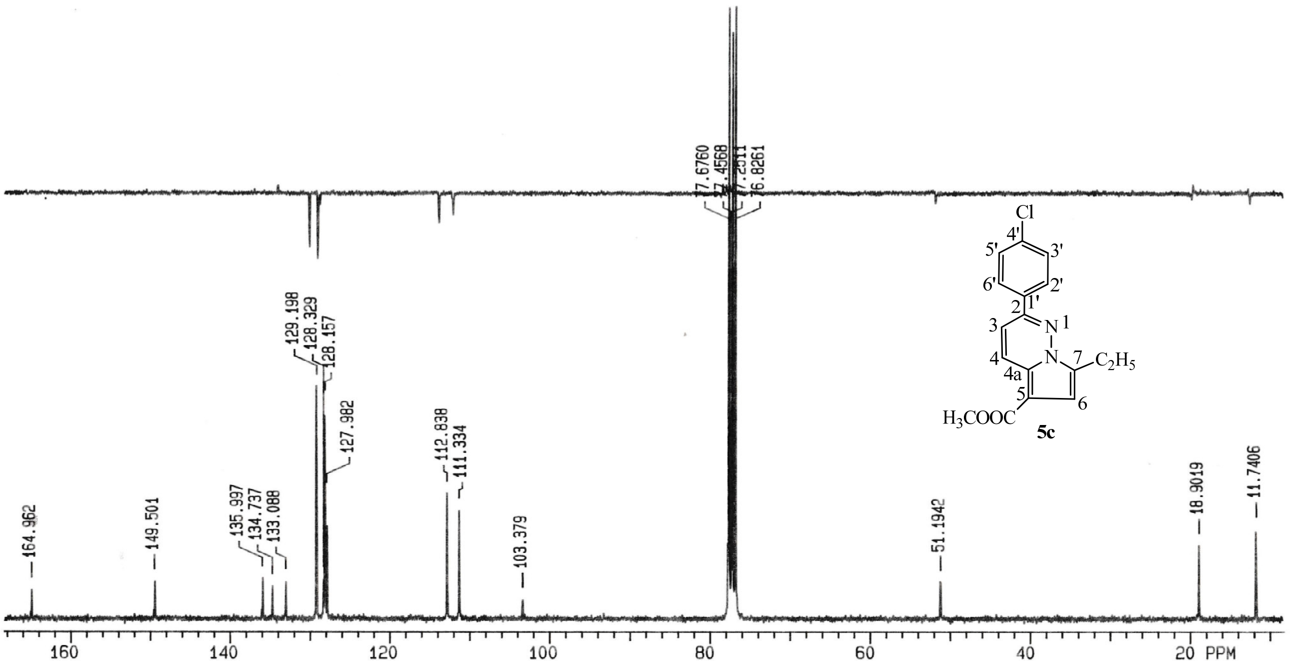

Figure 30S. The ^{13}C-NMR spectrum of **5c**

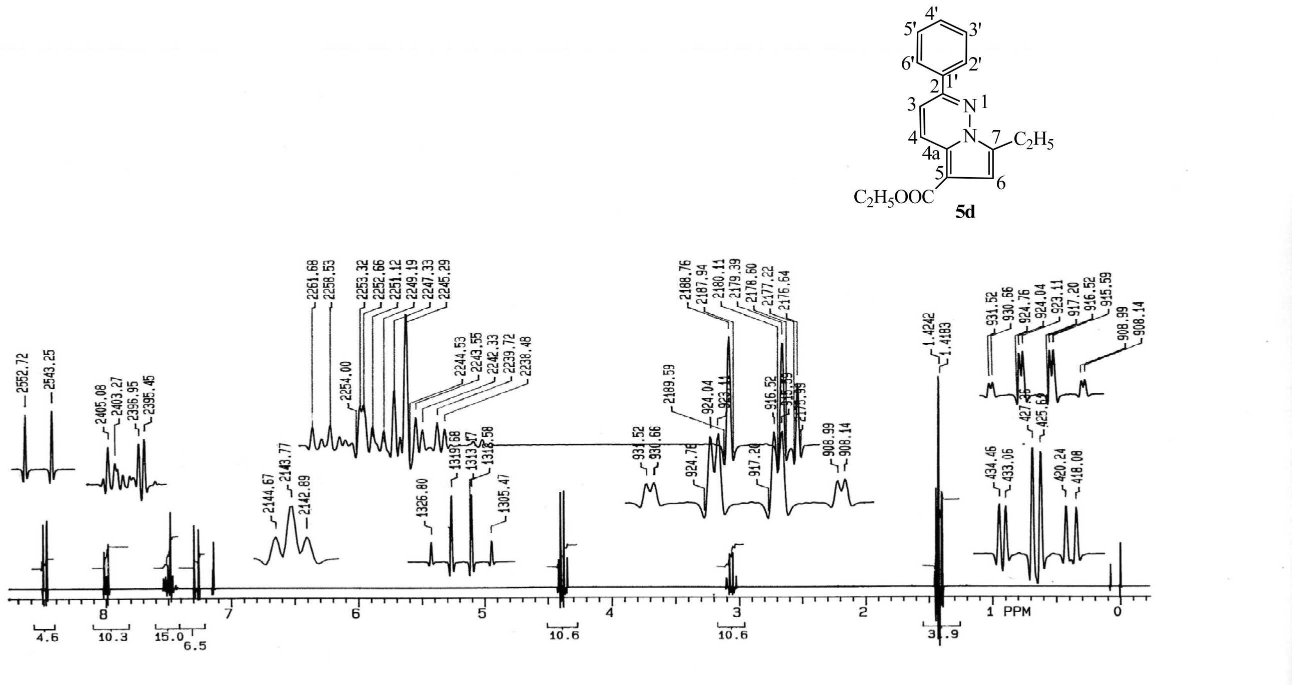

Figure 31S. The ^{1}H-NMR spectrum of **5d**

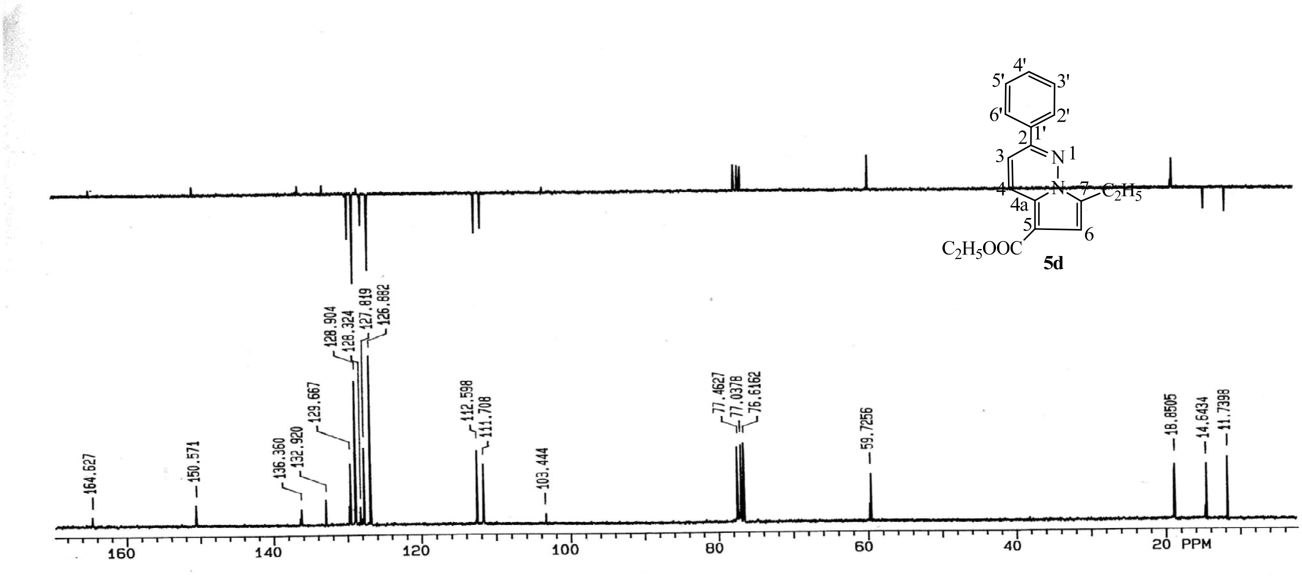

Figure 32S. The ^{13}C-NMR spectrum of **5d**

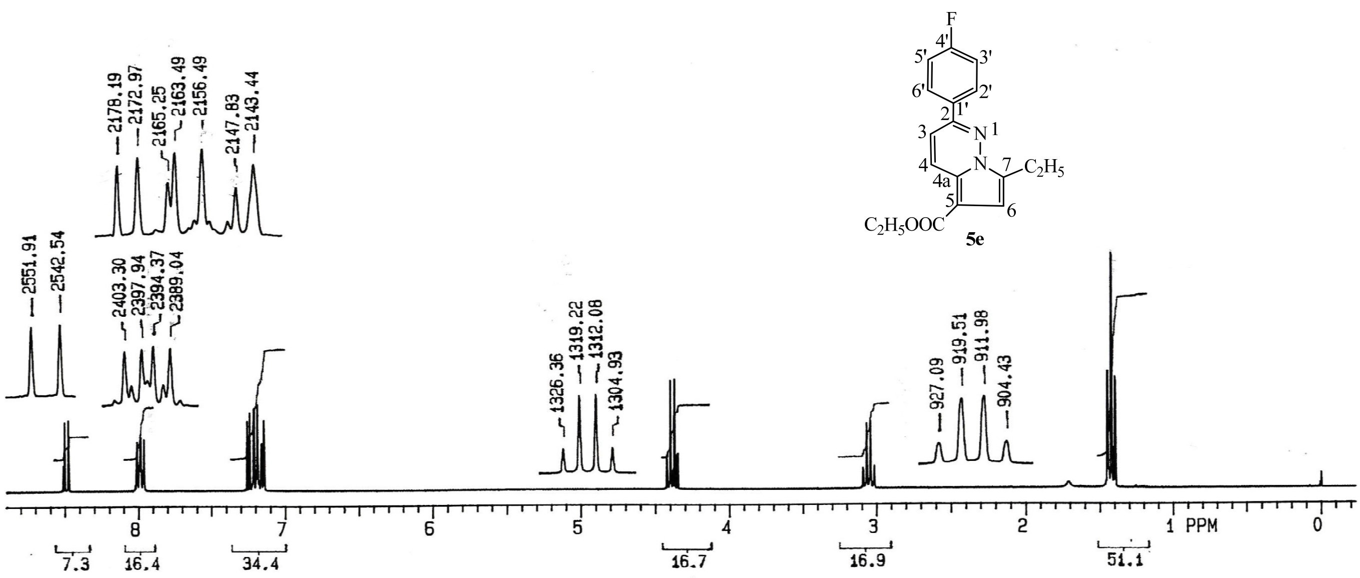

Figure 33S. The ^{1}H-NMR spectrum of **5e**

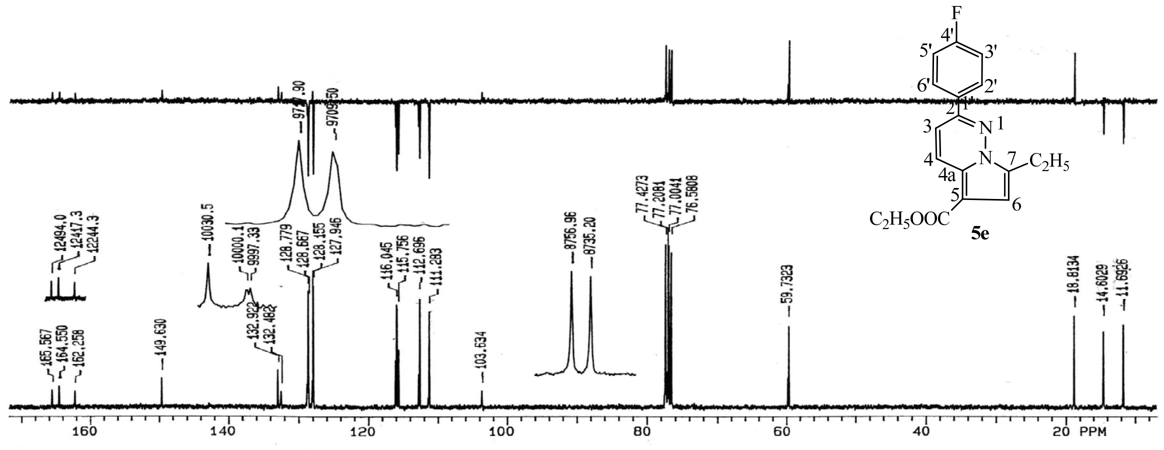

Figure 34S. The ^{13}C-NMR spectrum of **5e**

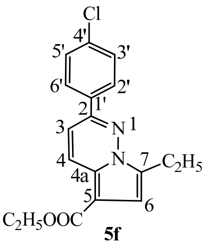

5f

Figure 35S. The ^{1}H-NMR spectrum of **5f**

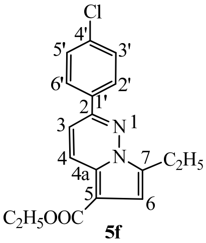

5f

Figure 36S. The ^{13}C-NMR spectrum of **5f**

3. X-ray data

Table 1S. Bond distances (Å) and angles (°).

Compound **1c**

Cl1-C4	1.739(3)

Copyright: © 2023 by the authors. Licensee MDPI, Basel, Switzerland. This article is an open access article distributed under the terms

and conditions of the Creative Commons Attribution (CC BY) license (https://creativecommons.org/licenses/by/4.0/).

O1-C12	1.333(3)
O1-C13	1.468(3)
O2-C12	1.200(3)
O3-C10	1.235(3)
N1-N2	1.355(3)
N1-C10	1.398(3)
N1-C11	1.472(3)
N2-C7	1.309(3)
C1-C2	1.395(3)
C1-C6	1.389(4)
C1-C7	1.481(3)
C2-C3	1.379(4)
C3-C4	1.378(4)
C4-C5	1.386(3)
C5-C6	1.388(4)
C7-C8	1.436(3)
C8-C9	1.336(3)
C9-C10	1.431(4)
C11-C12	1.526(3)
C11-C15	1.526(3)
C13-C14	1.491(4)
C15C16	1.520(4)

C12-O1-C13	114.9(2)
N2-N1-C10	125.1(2)
N2-N1-C11	117.86(18)
C10-N1-C11	116.8(2)
C7-N2-N1	118.40(19)
C2-C1-C7	121.1(2)
C6-C1-C2	117.9(2)
C6-C1-C7	121.0(2)
C3-C2-C1	121.5(3)
C4-C3-C2	119.6(2)
C3-C4-Cl1	120.52(19)
C3-C4-C5	120.5(2)
C5-C4-Cl1	118.9(2)
C4-C5-C6	119.3(3)
C5-C6-C1	121.2(2)
N2-C7-C1	116.5(2)
N2-C7-C8	121.3(2)
C8-C7-C1	122.2(2)
C9-C8-C7	119.6(3)

C8-C9-C10	121.1(2)
O3-C10-N1	119.3(2)
O3-C10-C9	126.4(2)
N1-C10-C9	114.3(2)
N1-C11-C12	108.2(2)
N1-C11-C15	112.7(2)
C12-C11-C15	110.26(19)
O1-C12-C11	111.5(2)
O2-C12-O1	124.9(2)
O2-C12-C11	123.5(2)
O1-C13-C14	107.1(2)
C16-C15-C11	111.4(2)

Compound **2b**

F1-C8	1.336(6)
O1-C12	1.307(8)
O2-C12	1.190(8)
O3-C1	1.247(8)
N1-N2	1.344(7)
N1-C1	1.388(9)
N1-C11	1.477(8)
N2-C4	1.312(9)
C1-C2	1.431(9)
C2-C3	1.323(10)
C3-C4	1.429(10)
C4-C5	1.484(8)
C9-C8	1.39
C9-C10	1.39
C8-C7	1.39
C7-C6	1.39
C6-C5	1.39
C5-C10	1.39
C11-C12	1.544(10)
C11-C13	1.508(10)
C13-C14	1.538(10)

N2-N1-C1	126.3(6)
N2-N1-C11	115.3(5)
C1-N1-C11	118.4(6)

C4-N2-N1	118.1(6)
O3-C1-N1	120.1(6)
O3-C1-C2	126.4(7)
N1-C1-C2	113.5(6)
C3-C2-C1	121.2(7)
C2-C3-C4	120.2(7)
N2-C4-C3	120.6(7)
N2-C4-C5	116.2(6)
C3-C4-C5	123.3(6)
C7-C8-C9	120
C8-C7-C6	120
C5-C6-C7	120
C10-C5-C6	120
C5-C10-C9	120
C8-C9-C10	120
F1-C8-C9	119.9(5)
F1-C8-C7	120.1(5)
C6-C5-C4	121.2(4)
C10-C5-C4	118.8(4)
N1-C11-C12	110.2(6)
N1-C11-C13	112.7(5)
C13-C11-C12	111.6(6)
O1-C12-C11	115.0(6)
O2-C12-O1	124.8(7)
O2-C12-C11	120.1(6)
C11-C13-C14	113.0(7)

Compound **5a**

O2A-C16A	1.346(2)
O2A-C17A	1.4419(19)
O1A-C16A	1.216(2)
N1A-N2A	1.3582(18)
N1A-C13A	1.389(2)
N1A-C10A	1.394(2)
N2A-C7A	1.324(2)
C14A-C13A	1.486(2)
C14A-C15A	1.520(2)
C16A-C11A	1.453(2)
C1A-C2A	1.382(2)
C1A-C6A	1.391(2)
C3A-C2A	1.385(3)
C3A-C4A	1.383(3)

C13A-C12A	1.371(2)
C12A-C11A	1.411(2)
C9A-C10A	1.405(2)
C9A-C8A	1.358(2)
C10A-C11A	1.393(2)
C5A-C4A	1.386(2)
C5A-C6A	1.394(2)
C8A-C7A	1.424(2)
C7A-C6A	1.483(2)
O1B-C16B	1.216(2)
O2B-C16B	1.351(2)
O2B-C17B	1.4365(19)
N1B-N2B	1.3598(19)
N1B-C10B	1.395(2)
N1B-C13B	1.383(2)
N2B-C7B	1.323(2)
C16B-C11B	1.456(2)
C2B-C3B	1.379(3)
C2B-C1B	1.381(2)
C11B-C10B	1.393(2)
C11B-C12B	1.413(2)
C6B-C7B	1.489(2)
C6B-C1B	1.395(2)
C6B-C5B	1.392(2)
C7B-C8B	1.420(2)
C10B-C9B	1.405(2)
C8B-C9B	1.360(2)
C12B-C13B	1.372(2)
C3B-C4B	1.378(3)
C14B-C15B	1.519(2)
C14B-C13B	1.485(2)
C4B-C5B	1.388(2)

C16A-O2A-C17A	115.91(13)

N2A-N1A-C13A	122.97(14)
N2A-N1A-C10A	126.26(14)
C13A-N1A-C10A	110.77(14)
C7A-N2A-N1A	115.51(14)
C13A-C14A-C15A	112.66(15)
O2A-C16A-C11A	111.83(15)
O1A-C16A-O2A	122.49(16)
O1A-C16A-C11A	125.68(17)
C2A-C1A-C6A	120.76(18)
C4A-C3A-C2A	119.61(18)
N1A-C13A-C14A	120.47(15)
C12A-C13A-N1A	106.09(15)
C12A-C13A-C14A	133.44(17)
C13A-C12A-C11A	109.49(16)
C8A-C9A-C10A	118.99(16)
N1A-C10A-C9A	116.29(15)
C11A-C10A-N1A	106.04(15)
C11A-C10A-C9A	137.67(15)
C4A-C5A-C6A	120.91(17)
C12A-C11A-C16A	127.86(16)
C10A-C11A-C16A	124.45(16)
C10A-C11A-C12A	107.61(15)
C1A-C2A-C3A	120.38(18)
C9A-C8A-C7A	120.14(17)
C3A-C4A-C5A	119.99(19)
N2A-C7A-C8A	122.82(16)
N2A-C7A-C6A	114.47(15)
C8A-C7A-C6A	122.71(16)
C1A-C6A-C5A	118.33(16)
C1A-C6A-C7A	120.50(16)
C5A-C6A-C7A	121.15(16)
C16B-O2B-C17B	115.78(13)
N2B-N1B-C10B	126.13(14)
N2B-N1B-C13B	122.93(14)
C13B-N1B-C10B	110.92(14)
C7B-N2B-N1B	115.57(14)
O1B-C16B-O2B	122.77(16)
O1B-C16B-C11B	125.69(17)
O2B-C16B-C11B	111.53(15)
C3B-C2B-C1B	120.54(18)
C10B-C11B-C16B	124.37(16)
C10B-C11B-C12B	107.47(15)

C12B-C11B-C16B	128.14(16)
C1B-C6B-C7B	120.26(16)
C5B-C6B-C7B	121.80(16)
C5B-C6B-C1B	117.94(17)
N2B-C7B-C6B	114.65(15)
N2B-C7B-C8B	122.82(16)
C8B-C7B-C6B	122.53(16)
N1B-C10B-C9B	116.27(15)
C11B-C10B-N1B	106.02(15)
C11B-C10B-C9B	137.71(16)
C9B-C8B-C7B	120.29(17)
C13B-C12B-C11B	109.42(16)
C4B-C3B-C2B	119.61(18)
C8B-C9B-C10B	118.89(16)
C13B-C14B-C15B	112.83(15)
C2B-C1B-C6B	120.82(18)
C3B-C4B-C5B	120.08(19)
C4B-C5B-C6B	121.00(18)
N1B-C13B-C14B	121.06(15)
C12B-C13B-N1B	106.16(14)
C12B-C13B-C14B	132.68(17)

Disclaimer/Publisher's Note: The statements, opinions and data contained in all publications are solely those of the individual author(s) and contributor(s) and not of MDPI and/or the editor(s). MDPI and/or the editor(s) disclaim responsibility for any injury to people or property resulting from any ideas, methods, instructions or products referred to in the content.
